# Academic and Social Impact of Menstrual Disturbances in Female Medical Students: A Systematic Review and Meta-Analysis

**DOI:** 10.3389/fmed.2022.821908

**Published:** 2022-02-15

**Authors:** Sabyasachi Maity, Jadzia Wray, Tamara Coffin, Reetuparna Nath, Shreya Nauhria, Ramsagar Sah, Randall Waechter, Prakash Ramdass, Samal Nauhria

**Affiliations:** ^1^Department of Physiology, Neuroscience, and Behavioral Sciences, St. George's University School of Medicine, St. George's, Grenada; ^2^Medical Student Research Institute, St. George's University School of Medicine, St. George's, Grenada; ^3^Department of Educational Services, St. George's University, St. George's, Grenada; ^4^Department of Psychology, University of Leicester, Leicester, United Kingdom; ^5^Sagar Hospital, Bangalore, India; ^6^Department of Public Health and Preventive Medicine, St. George's University School of Medicine, St. George's, Grenada; ^7^Department of Pathology, St. Matthew's University School of Medicine, Grand Cayman, Cayman Islands

**Keywords:** menstrual disturbances, pre-menstrual syndrome, pre-menstrual dysphoric disorder, dysmenorrhea, prevalence, medical students, undergraduate students

## Abstract

**Background:**

The stressful academic schedule of medical students poses an obvious challenge to their daily lifestyle. Psychosomatic discomfort poses a significant risk for inaccurate self-medication for ameliorating menstrual complications and feeling better, thus directly impacting personal and academic wellbeing.

**Objective:**

The impact of menstrual disturbances on academic life is not extensively explored. Therefore, the primary objective of this research was to probe the prevalence of menstrual disturbances and assess the academic and social impact. Finally, the authors provide an overview of pharmacological and other interventions students adopt to reduce clinical symptoms.

**Methods:**

A database search was conducted from the year 2016 till September 2021 for the studies reporting the prevalence of menstrual disorders in all geographic locations of the world. Keywords used for searching databases included “menstrual disturbances” and “medical students,” “prevalence” OR “symptoms” of “Premenstrual syndrome” OR “Premenstrual dysphoric disorder” OR “Dysmenorrhea” in medical students. Prospero Preferred Reporting Items for Systematic Reviews and Meta-Analyses (PRISMA) and Meta-Analysis of Observational Studies in Epidemiology (MOOSE) protocols were followed. The protocol was registered in the International prospective register of systematic reviews (PROSPERO), the Center for Reviews and Dissemination, University of York (CRD42021277962). The quality of the methodologies used in selected studies was evaluated by a modified version of Newcastle Ottawa Scale (NOS).

**Results:**

Initially, 1527 articles were available. After a review, 26 papers were selected for analysis. A total of 25 citations were identified for quantitative analyses, out of which 16 studies reported Pre-menstrual syndrome, 7 reported Pre-menstrual dysphoric disorder, and 13 articles reported dysmenorrhea. The pooled prevalence of Pre-menstrual syndrome was 51.30%, Pre-menstrual dysphoric disorder was 17.7%, and dysmenorrhea was 72.70%. Most common associated lifestyle factors were stress, excessive caffeine intake and lack of exercise. Painkillers, hot packs and hot beverages were amongst the common measures taken by the students to relieve their symptoms.

**Conclusions:**

The current situation calls for action to accommodate students' needs and bridge the social gap regarding menstrual health. Proactive measures by medical educators and stakeholders are required for an inclusive, accommodating educational environment which will minimize the gender discrepancy in academic satisfaction and professional life.

## Introduction

Menstruation has a significant impact on a woman's physical, mental and social wellbeing. Menstrual health is defined as “a state of complete physical, mental, and social wellbeing and not merely the absence of disease or infirmity, in relation to the menstrual cycle” (1). Between menarche and menopause most women mensurate, thus menstrual health plays and integral part in women's overall heath. Cyclic discharge of blood from uterine corpus between menarche and menopause is usually how mensuration is defined. Bleeding lasts between 2 and 7 days during each cycle of 21–31 days, average 28 days. Almost 70–80% of women does not experience any disruption in carrying out their normal daily activities during or days leading to mensuration, while remaining 20–30% experiences Pre-menstrual Syndrome (PMS). Three to eight percent women experiences severe symptoms. PMS manifests as physical and emotional symptoms that occurs 1–2 weeks before actual menstruation. Common symptoms of PMS include physical symptoms such as abdominal bloating and cramp, extreme fatigue, tiredness, tendered breast and headaches; emotional symptoms like mood swings, irritability, depression, hyperphagia, and forgetfulness and difficulty with concentration (2–5).

Pre-menstrual dysphoric disorder (PMDD) is a severe form of PMS that occurs in 3–8% females resulting in serious psychological symptoms (6). PMDD is listed as pre-menstrual syndrome/pre-menstrual tension/pre-menstrual tension syndrome in the International Classification of Diseases eleventh version (ICD-11), whereas the Diagnostic and Statistical Manual of Mental Disorders (DSM-V) and American College of Obstetricians and Gynecologists (ACOG) recognize it as pre-menstrual dysphoric disorder (7, 8). According to DSM-5 a female must possess 5 out of 11 symptoms to be categorized as PMDD, and symptom of mood swings must be present for diagnosis. Symptoms include irritability, depression, and excessive tension. These symptoms are often so severe and can lead to dysfunction and poor quality of life. The etiology of PMDD is still unknown, and studies show that being unmarried, mental distress, menstrual dysfunction and poor health can all lead to PMDD (3, 4, 9). However, there is no reported difference between women of different socio-economic status and culture (10).

Many forms of treatment and self-care are available to control the symptoms of most women, but 3 in 4 women suffer from some form of PMDD (11). Another common menstrual disorder is Dysmenorrhea (primary and secondary), which refers to pain during menstruation. Unlike primary dysmenorrhea, secondary dysmenorrhea is associated with pelvic pathology. The prevalence of dysmenorrhea has been reported to range from 15.8 to 89.5%, with ~20% experiencing condition as severe (12). Reproductive health and women's health in general is not sufficiently represented in basic and translational research. Millions of females are catastrophically affected by menstruation in terms of mental, physical, and social health. Surprisingly, previous research has shown up to 40% of women quit work and stayed home due to abnormal menstrual bleeding, thus leading to poor professional and personal life quality (13). According to a recent Lancet article all those who menstruate are being denied basic human rights, thus shaping United Nations development goals. These goals include poverty, education, health, water and sanitation, and gender equality. Addressing menstrual health problems is crucial in reaching these goals (14). It is now well-documented that educating women has a significant contribution to a more stable and resilient society, which provides all individuals equal opportunity to attain their full potential (15). Effects of menstruation on academics have been studied throughout the century. A recent systematic review established the relationship between dysmenorrhea and academic impairment such as absenteeism, lesser participation in classroom activities, lack of concentration, and degrading academic performance (16). Female students generally prefer not to seek medical attention or self-medicate using over-the-counter pain killers or other methods to control menstrual pain (6, 10, 17).

Studies suggests that female medical students around the world are also disadvantaged due to abnormal menstrual cycles. PMS, PMDD, and dysmenorrhea are leading causes of academic and personal wellbeing impairment in a medical school (3, 18–21). Moderate to severe psychosomatic menstrual symptoms are reported in the medical and health sciences students (2, 22–25).

The absence from classes and other social activities during abnormal menstruation creates anticipatory anxiety and up to 20% medical students loathe menstruation due to severe pain (21, 26, 27). A cross-sectional study on female medical students suggested 39.4% prevalence of PMS, with 14.2% reporting severe PMS. Quality of life score was reported be low in half of the students (28). The implication of abnormal menstrual cycles in female medical students is alarming. Females are more prone to depression and related symptoms compared to men (29). Abnormal menstruation further adds to the existing high stress among the medical students due to voluminous curricular content (30–33). A study of 414 medical students reported positive correlation between PMS and anxiety, depression, and stress. 11.8% students reported moderate, whereas 1.7% reported severe depression (34).

Although a few systematic reviews addressed the prevalence of menstrual disturbances among the female population (10, 17, 35), no such attempts were made in the last 5 years to explore the impact of problematic menses on the quality of life by providing evidence with meta-analysis of published studies. Thus, present research explores intrinsic inequality in the medical school curriculum, which situate female medical students a step backwards from the beginning of their academic life because of abnormal physiology. Furthermore, the current paper provides an insight on epidemiological details of menstrual disturbances.

Over time, medical education has reformed itself to accommodate students' needs by including students with learning disabilities (31, 32). An inclusive medical school curriculum considering the students with any menstrual disturbance is essential for equality (18). Medical educators are in an advantageous position to reframe the negative attitude or eliminate the negative emotions associated with menstruation in medical students. It is time for medical educators to address the gender divide regarding access to education resources and better-quality life (36). Due to lack of awareness and initiatives among medical educators there is still have a long way to go to address this issue. Keeping up with the opposite gender and not experiencing equality can be detrimental, the psychosomatic complications of menstrual disturbances, thus, are a significant concern for female medical students in the current competitive world where gender equality has seen great uprising (14, 18, 31, 32, 37, 38). Thus, the aims of current study are-

a) Probe the prevalence of PMS, PMDD and dysmenorrhea using meta-analysis among medical students.
b) To assess the effect of this prevalence on class absenteeism and overall quality of life.
c) To provide an overview of pharmacological and other modes of interventions adopted by female medical students worldwide to reduce the clinical symptoms of menstrual disturbances.

## Materials and Methods

Preferred Reporting Items for Systematic Reviews and Meta-Analyses (PRISMA) and Meta-Analysis of Observational Studies in Epidemiology (MOOSE) protocols were followed by the researchers (39, 40). The protocol used in this study was registered in International prospective register of systematic reviews (PROSPERO), the Center for Reviews and Dissemination, University of York (CRD42021277962) prior to the commencement of the project.

### Search Strategy

Published studies *were searched* in electronic databases namely, PubMed (US National Library of Medicine, National Institutes of Health), Scopus, Embase, and Web of Science for potentially relevant studies from inception up to September 2021. Articles published in English from selected databases were included. The authors were required to reach a consensus among themselves on the final search strategy. The medical subject headings (MeSH) search terms included “menstrual disorder,” “Premenstrual Syndrome” and “Premenstrual Dysphoric Disorder” including all subheadings. The following search strategy was used to identify studies in PubMed: (“Menstruation Disturbances” [Mesh]) AND “Students” [Mesh]. Using simple search terms “Menstruation Disturbances AND Students,” search was conducted through PubMed in addition to our MESH search. For Scopus, the following search strategy was used: ALL (Menstruation disturbances and students) AND [LIMIT-TO (DOCTYPE, “ar”) OR LIMIT-TO (DOCTYPE, “re”)] AND [LIMIT-TO (LANGUAGE, “English”)]. For EMBASE, we used: (“menstruation disorder”/exp OR “menstruation disorder”) AND “medical students.” Finally, the relevant articles were also included by adopting the snowball method which involves searching the bibliographic list of selected articles.

### Selection of Studies

Two independent reviewers (SM and SN) screened the retrieved papers based on titles and abstracts. Criteria for examination of full text of the relevant paper after the initial database screening were as follows:

- Articles reporting data on impact and prevalence of PMS, PMDD, and/or dysmenorrhea that could be extracted for statistical analysis were only included
- Studies conducted in any geographical location but on medical/health sciences/nursing students
- All the studies that include cross-sectional studies, or cohort based
- Studies published from 2016 till September 2021 (last 5 years) and the female population studied adequately
- The non-peer-reviewed editorials, letters, commentaries, incomplete data, reviews, conference posters, preprints, and thesis were excluded

**Figure 1:**
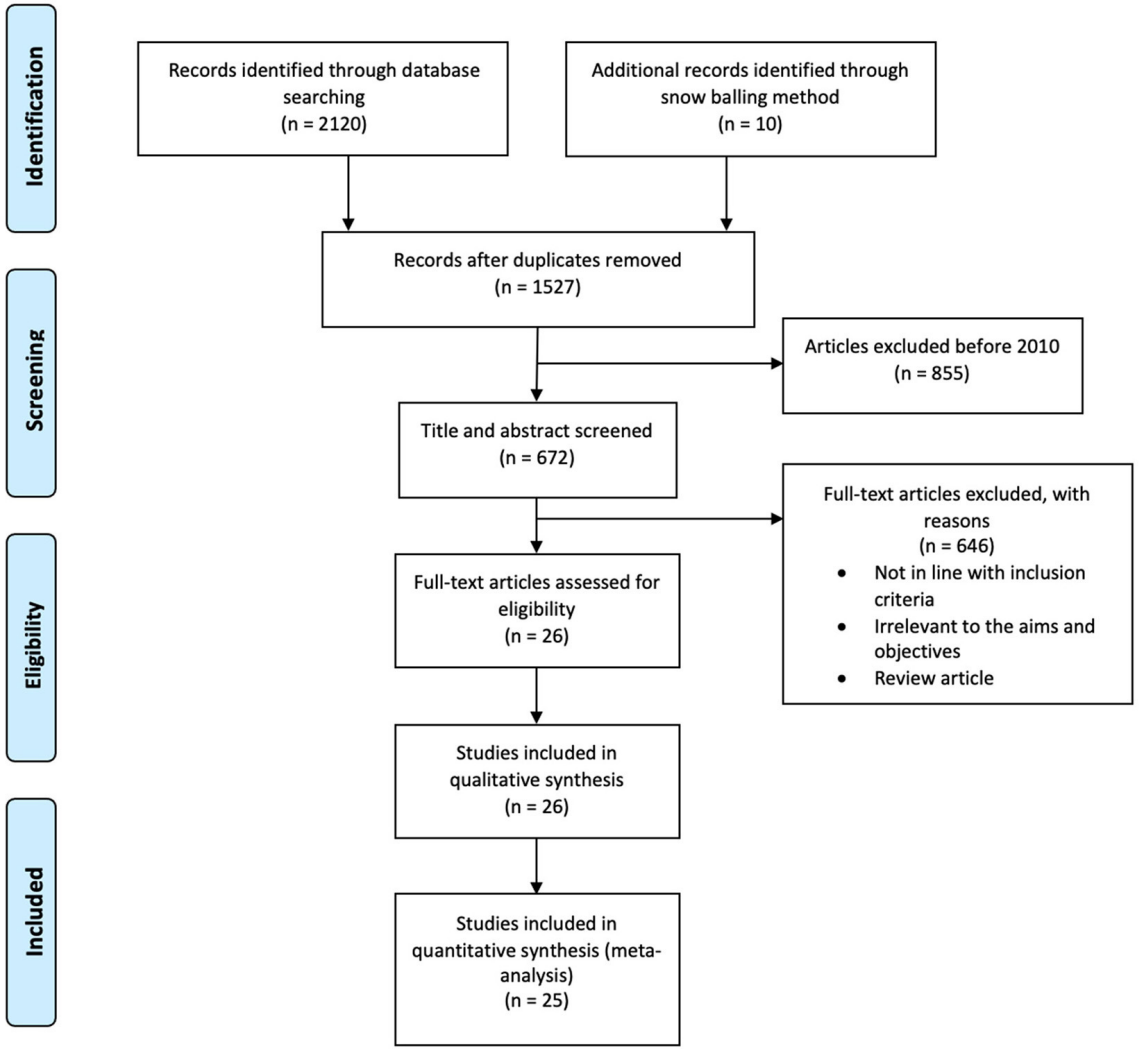
PRISMA protocol of literature search process.

Any confusion or doubts regarding the study selection were resolved by reaching a consensus. Figure 1 represents the process of study selections for the systematic review and meta-analysis as per the PRISMA protocol.

### Quality Assessment

Two independent reviewers (SM and SaN) evaluated the quality of the methodologies used in selected studies by a modified version of Newcastle Ottawa Scale (NOS) (41). Five segments of the NOS scale were included with offering of one (^*^) to each with a positive evaluation regarding any of the assigned point as per the criteria. The five segments for assessing quality of each study article were: “sample representativeness,” “sample size,” “ascertainment of any of the inclusion menstrual disorder,” “comparability between respondents and nonrespondents,” and “statistical quality.” According to the resultant number of points assigned, each study was judged to be at high (≥3 ^*^) or low (<3 ^*^) quality. Any discrepancies concerning the author's judgments were resolved by consensus and by discussing with another reviewer PR. Supplementary Table 1 represents the raw data of quality assessment using the modified NOS.

### Data Extraction

Author SM extracted the relevant data, and the data was crosschecked by SaN and others. In a blank excel sheet, data on author and year of publication, geographical location, duration of the study, age range of the study participants, total number of populations, diagnosis and scale used as diagnosis criteria, and reported prevalence for each eligible study were extracted. The included study authors were contacted for clarification and assistance with incomplete study and non-response was considered as exclusion. Any disagreement amongst authors was resolved by consensus and discussion with PR.

### Data Analysis

Meta-analysis of quantitative data was performed to estimate the cumulative prevalence from individual studies. The summary estimates of prevalence were reported along with their 95% confidence intervals (CI) for PMS, PMDD, and dysmenorrhea. The pooled prevalence data was presented in a Forest plot. All the analyses were done using “Comprehensive meta-analysis” software version 3. Presence of publication bias was examined by the visual inspection of funnel plot. The “one-study-removed” procedure was used as a sensitivity analysis to determine whether the overall estimates of menstrual disorders in female medical students were influenced by outlier studies.

## Results

### Search Results and Study Characteristics

Our search through the databases finally identified 26 articles on prevalence of menstrual disturbances including PMS, PMDD, dysmenorrhea that were included in the systematic review from 2016 till 2021. The article exclusion criteria included the following reasons:

- Not relevant to the objective
- Not in line with the inclusion criteria
- The studies were not conducted on medical students
- The full text pdf was not available
- Not original research
- No availability of statistical results

All the included studies were of cross-sectional design across the world at varied number and time frames. Although most of the studies were on medical students however, a few reported prevalence from non-medical students too with a reasonable statical comparison of prevalence. The total number of female medical student from the included studies was 4,874. Although most of the studies exclusively reported the prevalence of either PMS or PMDD or dysmenorrhea however, a few studies do indicate prevalence of two menstrual disturbances in a single population. Therefore, the prevalence data were calculated accordingly. A detailed synthesis of included studies is provided in Table 1.

**Table 1:** Qualitative synthesis of the 26 included studies.

**References**	**Place**	**Study design/data collection format**	**Data collection period**	**Population, age (years)**	**Reports on/scale used**	**Population studied (n)**	**Prevalence of PMS No. (%)**	**Prevalence of PMDD No. (%)**	**Dysmenorrhea No. (%)**
Alkhamis et al. (3)	Saudi Arabia	Cross sectional	Not indicated	Female medical student, 20–23	PMS, PMDD/self-administered questionnaire	258	29 (11.2%)	32 (12.5%)	Not indicated adequately
Kushwaha et al. (21)	Nepal	Cross sectional	October 2019 to December 2019	Female medical student, 17–25	Primary dysmenorrhea/verbal multidimensional scoring system (VMSS)	75	Not indicated adequately		Mild primary dysmenorrhea is prevalent in 42 (49.4%) MBBS students and moderate to severe primary dysmenorrhea is prevalent in 33 (56.9%) MBBS students
Al-Shahrani (12)	Saudi Arabia	Cross sectional	Not indicated	Female medical student, 18–25	PMS/The Premenstrual Syndrome Scale (PSS)	388	252 (64.9%)	Not indicated adequately	152 (39.2%)
Hashim et al. (4)	Saudi Arabia	Cross sectional	September 2017 and May 2018	Female medical student, 19–22.4	Primary dysmenorrhea/SF-36	336	Not indicated adequately	Not indicated adequately	269 (80.1%)
Shah and Christian (5)	India	Cross sectional	Not indicated	Female medical student, 18–24	PMS and PMDD/Premenstrual Symptoms Screening Tool	166	31 (18.9%) had moderate to severe PMS	10 (6.09%)	Not indicated adequately
Kanti et al. (42)	India	Cross sectional	July to August 2019	Female medical student, 21–23	PMS and Dysmenorrhea/Menstrual symptom questionnaire and menstrual bleeding questionnaire	150	86 (56%)	Not indicated adequately	86 (56%)
Minichil et al. (9)	Ethiopia	Cross sectional	May to June, 2019	Female medical student, 18–26	PMDD/DSM-5	386	Not indicated adequately	134 (34.7%)	Not indicated adequately
Bilir et al. (43)	Turkey	Cross sectional	December 2017 and January 2018	Female medical student, 18–27	PMS and Dysmenorrhea	50	Not indicated adequately	Not indicated adequately	12 (26%)
Özder and Salduz (44)	Turkey	Cross sectional	May 2017 to June 2017	Female medical student, 17–26	Dysmenorrhea/structured questionnaires to state socio-demographic and medical characteristics, and their dysmenorrheal status and habits, and Visual analog scale to assess the severity of dysmenorrhea	413	Not indicated adequately	Not indicated adequately	329 (79.7%)
Verma et al. (2)	India	Cross sectional	Within 1 weeks' time	Female medical student, 17–28	PMS and Dysmenorrhea	183	156 (85.24%)	Not indicated adequately	111 (60.66%)
Majeed-Saidan et al. (34)	Pakistan	Cross sectional	December 2017 to May 2018	Female medical student, 12–51	PMS/ACOG PMS diagnostic criteria	280 of them were medical students	Moderate PMS: 166(59.4%). Severe PMS 22 (8%). But no differentiation for medical students.	Not indicated adequately	Not indicated adequately
Nama et al. (45)	India	Cross sectional	June 1, 2020 to July 31, 2020	Female medical student, 19–25	PMS and Dysmenorrhea/pre tested, structured, self-administered questionnaire	100	83 (83%)	Not indicated adequately	86 (86%)
Zalat et al. (46)	Saudi Arabia	Cross sectional	Academic year [2017–2018]	Female medical student, 21–23	PMS/Premenstrual Evaluating Questionnaire (PEQ) based on the criteria of the American college of obstetrics & gynecology (ACOG) for the diagnosis of PMS	98	54 (55.10 %)	Not indicated adequately	Not indicated adequately
Yadav and Taneja (47)	India	Cross sectional	Not indicated	Female medical student, 17–22	PMS and Dysmenorrhea/A self-descriptive cross-sectional study	200	64 (32%)	Not indicated adequately	140 (70%)
Sharma et al. (48)	India	Cross sectional	Not indicated	Female medical student, 18–20	PMS/A self-administered questionnaire	209	121 (57.9%)	Not indicated adequately	Not indicated adequately
Shamnani et al. (24)	India	Not indicated	Not indicated	Female medical student, 18–25	PMS and PMDD/diagnosis criteria proposed by American College of Obstetrician and Gynecology	240	156 (65%)	29 (12%)	Not indicated adequately
Rajkumari et al. (49)	India	Stratified random sample method	Not indicated	Female medical student, 18–22	PMS/Inventory to Measure Psychosocial Stress (IMPS) and menstrual questionnaire	81	65 (80%)	Not indicated adequately	Not indicated adequately
Ghaderi et al. (50)	Iran	Cross sectional	April 2013 to July 2013	Female medical student, 19–25	Dysmenorrhea/visual analog scale (VAS)	197	Not indicated adequately	Not indicated adequately	194 (98.4%)
Acikgoz et al. (51)	Turkey	Cross sectional	March to June 2016	Female medical student, 17–31	PMS/Premenstrual syndrome scale (PMSS)	100	52 (52%)	Not indicated adequately	Not indicated adequately
Rumana Akbari et al. (52)	India	Cross sectional	For a period of six months in 2015	Female medical student, 20–25	PMS/PMS self-evaluation questionnaire	270	84 (31.1%)	Not indicated adequately	Not indicated adequately
Katwal et al. (53)	Nepal	Cross sectional descriptive study	1st Dec. 2012 to 31st Jan. 2013	Female medical student, 16–24	Dysmenorrhea/questionnaire to complete	184	Not indicated adequately	Not indicated adequately	123 (67%)
Aryal et al. (25)	Nepal	Cross sectional	November to December 2015	Female medical student, 17–25	PMS and PMDD/American College of Obstetrics and Gynecology (ACOG) criteria. The diagnosis of PMDD was based on Diagnostic and Statistical Manual of Mental Disorders (DSM-V).	185	113 (61.1%)	72 (38.9%)	Not indicated adequately
Jaiprakash et al. (54)	Malyasia	Cross sectional descriptive study	March to May 2012	Female medical student, 17–30	PMS and Dysmenorrhea/questionnaire and verbal-multi-dimensional scoring system	215	153 (91.6%)	Not indicated adequately	167 (78%)
Goweda et al. (55)	Saudi Arabia	Cross sectional	During the academic year 2013/2014	Female medical student, Age not indicated	PMDD/Diagnostic and Statistical Manual of Mental Disorders, Fifth Edition	183	Not indicated adequately	67 (36.6%)	Not indicated adequately
Raval et al. (56)	India	Cross sectional	January to August, 2012	Female medical student, 17.3–20.5	PMS and PMDD/DSM-IV-TR criteria and SCID-PMDD	71	5 (7%)	1 (1.5%)	Not indicated adequately
Maryam et al. (57)	Indonesia	Cross sectional	September 2015	Female medical student, 19–22	Dysmenorrhea/DASS 42	136	Not indicated adequately	Not indicated adequately	74 (54.4%)
Total number of students						4,874			

### Quality Assessment of Included Studies Using NOS

The final included articles were predominantly of cross-sectional design. Therefore, a modified NOS was used as explained in the methods section. To evaluate each study, a (^*^) was assigned to the any of the criteria of the NOS. Only one study (34) was rated as low (<3 ^*^). Other included studies were rated as high. Table 2 represents the summary of quality assessment using modified NOS.

**Table 2:** Quality assessment of studies using modified Newcastle Ottawa Scale.

**Reference**	**Selection**		**Outcome**			**Total score**
	**Sample representativeness**	**Sample size**	**Response rate**	**Assessment of outcome**	**Statistical tests**	
Alkhamis et al. (3)	*	–	–	*	*	***
Kushwaha et al. (21)	*	*	–	*	*	****
Al-Shahrani (12)	*	–	–	*	*	***
Hashim et al. (4)	*	*	*	*	*	*****
Shah and Christian (5)	*	*	–	*	–	****
Kanti et al. (42)	*	–	–	*	*	***
Minichil et al. (9)	*	*	–	*	*	****
Bilir et al. (43)	*	*	*	*	*	*****
Özder and Salduz (44)	*	–	–	*	*	***
Verma et al. (2)	*	–	–	*	*	***
Majeed-Saidan et al. (34)	–	*	–	*	–	**
Nama et al. (45)	*	–	–	*	*	***
Zalat et al. (46)	*	–	–	*	*	***
Yadav and Taneja (47)	*	–	–	*	*	***
Sharma et al. (48)	*	*	–	*	*	****
Shamnani et al. (24)	*	–	–	*	*	***
Rajkumari et al. (49)	*	*	–	*	*	****
Ghaderi et al. (50)	*	*	–	*	*	****
Acikgoz et al. (51)	*	–	–	*	*	***
Rumana Akbari et al. (52)	*	–	–	*	*	***
Katwal et al. (53)	*	–	–	*	*	***
Aryal et al. (25)	*	–	–	*	*	***
Jaiprakash et al. (54)	*	–	–	*	*	***
Goweda et al. (55)	*	*	–	*	*	****
Raval et al. (56)	*	*	–	*	*	****
Maryam et al. (57)	*	–	–	*	*	***

SM and RN individually assessed the impact of menstrual disturbances and the lifestyle factor associations.

### Impairment of Academic and Social Life

The high prevalence of menstrual disturbances impacted the academic and social life. Students were missing classes and some reported lower grades compared to others. In addition, many reported impairments of various aspects of the quality of life such as meeting friends and co-workers, relationships with their family and partners, etc. What alarming is the intervention by the medical students to reduce the symptoms of menstrual disturbances. Most of the students reported to self-medicate by using painkillers such as NSAID's and other hot drinks. A very few consulted doctors for treatment. Some medical students preferred not to share the menstrual discomfort and perceived it as taboo. The details of academic and health impact of the respective studies and frequent measures adopted by the students are listed in Table 3.

**Table 3:** Academic and social impact of menstrual abnormalities and the adopted intervention by the medical students to reduce complications.

**Reference**	**Impact on academic and social life**	**Intervention to reduce menstrual disturbances/Conclusion**
E. G. Alkhamis et al. (3)	• One third of students was leaving early during the class • Indicated low-grade scoring • Reported lower grades than others	• Coffee (71.3%) and painkillers (57.4%) as the most common type of treatment.
Kushwaha et al. (21)	• Impairment of social and personal life	• Over two-thirds used home remedies alone or in combination with analgesic drugs. • Mefenamic acid was the most common self-medicated drug. • The majority had used drugs once a day and more than half had insufficient knowledge about drug dose.
Al-Shahrani (12)	• Menstruation significantly affected the related quality of life subscales concerning the homework interface • Stress increases to reading class material.	• Among the students who responded yes to PMS, only 4.1% use of drugs for menstrual regulation and 60% did not use any drug
Hashim et al. (4)	• More than half reported increase in their absenteeism. • More than 80% reported a decrease in their study time, participation, and concentration.	• Periodical awareness programs should be introduced to minimize the consequences
Shah and Christian (5)	• The school/work efficiency or productivity impaired • Home responsibilities and relationship with friends, classmates/coworkers and family impaired for majority with PMS. • All of the above functional impairments were observed in all with PMDD.	• Less than one-fourth were using heating pads for their cramps. Herbal tea was used for its soothing effects and preventing menstrual cramps for some. • A few were using any of the non-contraceptive hormonal medicines although more than 80% did not consume such products on a daily basis.
Kanti et al. (42)	• 18% remained absent from class for 1 or 2 days due to pain.	• Only 7.1% were using medication.
Minichil et al. (9)	• 35.5% perceived menstrual pain has an impact on their academic performance. • 72% missed their class at least once.	• Some self-medication with paracetamol and ibuprofen. Tea and coffee were consumed by 37 and 51%, respectively. • Only 7.1% consulted healthcare providers.
Bilir et al. (43)	18.2% reported school absenteeism due to PMS	• 92.6% used analgesic very commonly. Only 10% were on oral contraceptive pills. • Hot packs (63.4%), hot showers (56.6%), rest (34.8%), herbs (14.8%), exercise (9.7%), and hobbies (8.2%).
Özder and Salduz (44)	• 34% students skipped a class	• 44% felt the need for analgesics. Only 14% sought medical advice.
Verma et al. (2)	• 12 reported absenteeism from the college.	• More than 50% needed some form of medication
Majeed-Saidan et al. (34)	• The overall wellbeing is impaired.	• More than 50% used over-the-counter medications 76.8% used alternative therapy • 15% visited gynecological clinics and <1% visited psychiatric clinics • The most commonly used painkillers are acetaminophens (23.3%), and the least commonly used painkillers are oxicam derivatives (0.2%). • The usage of non-steroidal anti-inflammatory drugs (NSAIDs) is 11% “ineffective” while 29.6% reported it as “very effective,” 38% reported
Nama et al. (45)	• Decreased academic performance, difficulty in concentrating, forgetfulness, adjustment difficulties, loneliness	• Authors recommend inclusive and more flexible medical education curriculum design • Eliminating sedentary lifestyles
Zalat et al. (46)	• 35% reported forgetfulness and 40% reported confusion • 42% reported insomnia	• More than 80% did not have history of taking medical advice for PMS
Yadav and Taneja (47)	• 29% missed social activities and 12% missed college	• 55% needed drugs to treat menstrual disorders and 82.5% had misconceptions and taboos related to menstruation.
Sharma et al. (48)	• Reduced productivity and inability of participate in social activities for majority • Relationship with others affected for one fifth	• Authors discuss the association between caffeine intake and a higher PMS • Healthier lifestyles can positively impact social activity and interpersonal relations thus enhancing overall productivity
Shamnani et al. (24)	• 12% were absent in educational activities and 32% avoided joining social activities	• 45% of symptomatic participants consulted to their mothers, 28% to their friends, 21% to others. • Only 6% consulted to physician
Rajkumari et al. (49)	• Higher stress score	• Stress is a positive predictor for all menstrual disorders
Acikgoz et al. (51)	• Depression, fatigue, anxiety	• Depression risk should be evaluated in students with PMS
Ghaderi et al. (50)	• 76% had negative impact on daily activities and 35% were absent from class	• More than half used ibuprofen, diclofenac. Many preferred herbal tea, chamomile, ginger, hot pack, etc.
Rumana Akbari et al. (52)	• Not indicated adequately	• The prevalence of PMS is directly proportional to age and academic year of study. PMS was found to be more among students residing in hostels.
Katwal et al. (53)	29% missed classes	• Positive relationship between psychological stress and dysmenorrhea. • Dysmenorrhea is the leading cause of recurrent short-term school absence in young ladies; this issue certainly needs to be addressed.
Goweda et al. (55)	Difficulty in concentrating	• Improving early detection of PMDD and proper management can improve general wellbeing and ensure a better health • Addressing the current issue and accommodating students can enhance scholastic performance
Jaiprakash et al. (54)	• 32% had social life impairment and 22% were absent from college	• Most of them did not take any medications.
Aryal et al. (25)	Overall wellbeing is decreased	• Dysmenorrhea symptoms should be effectively screened by healthcare providers • Potentially lead to an improvement of academic performance and professional skills of students
Raval et al. (56)	• Majority had reduced school/work efficiency or productivity • Relationships with friends, classmates/co-workers and social life activities impaired	• Various screening and assessment tools are available such as PSST for PMS and SCID-PMDD • Regular use of such tools can identify and thus overall lead to an improvement of academic performance
Maryam et al. (57)	• Productivity decreased	• Stress management to prevent more severe dysmenorrhea, and increase the productivity

### Lifestyle Factors Associated With Menstrual Disturbances

**Figure 2:**
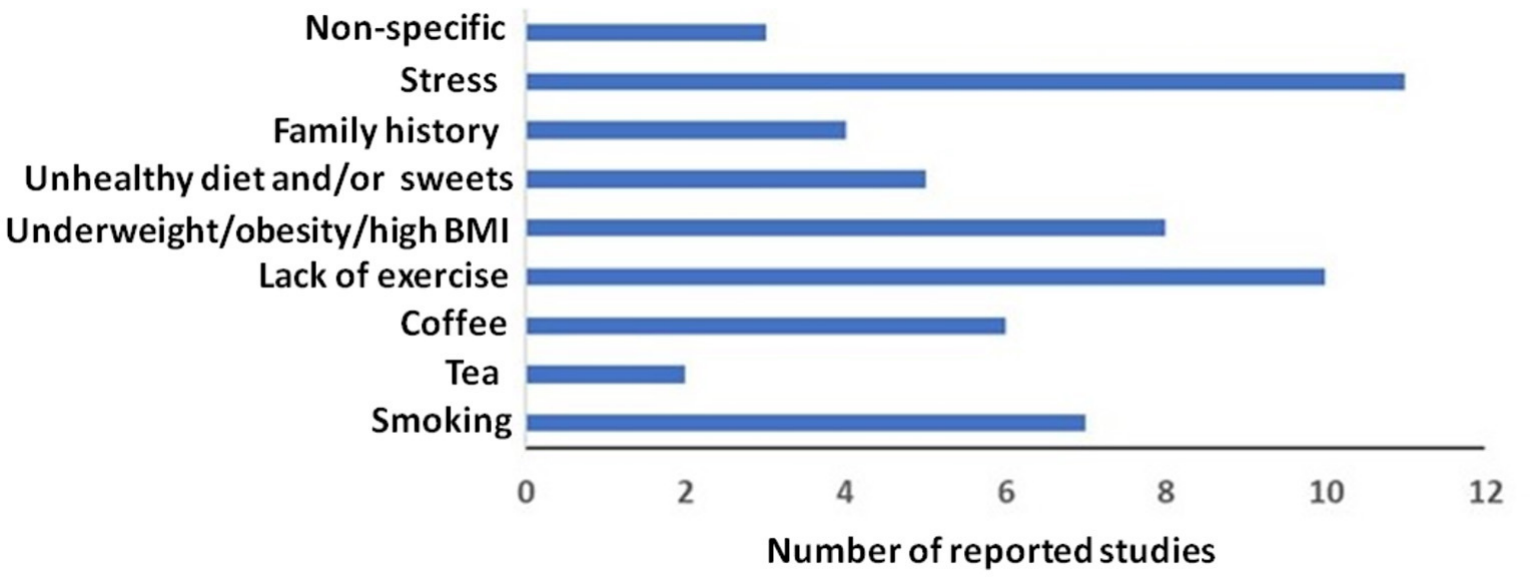
Major lifestyle factors associated with menstrual abnormalities.

The lifestyle factors associated with menstrual disturbances found in our study are shown in Supplementary Table 2 which represents some characteristics of everyday life which potentially lead to menstrual disturbances. For example, anticipatory stress or family history of other psychologic disorders are prone to have menstrual problems. Likewise, lack of exercise, excessive caffeine and tea intake, smoking, unhealthy dietary habits, and abnormal body weights are lifestyle factors associated with the development of menstrual complications. Figure 2 depicts the graphical representations of the factors leading to the menstrual abnormalities in medical students.

### Prevalence of PMS

**Figure 3:**
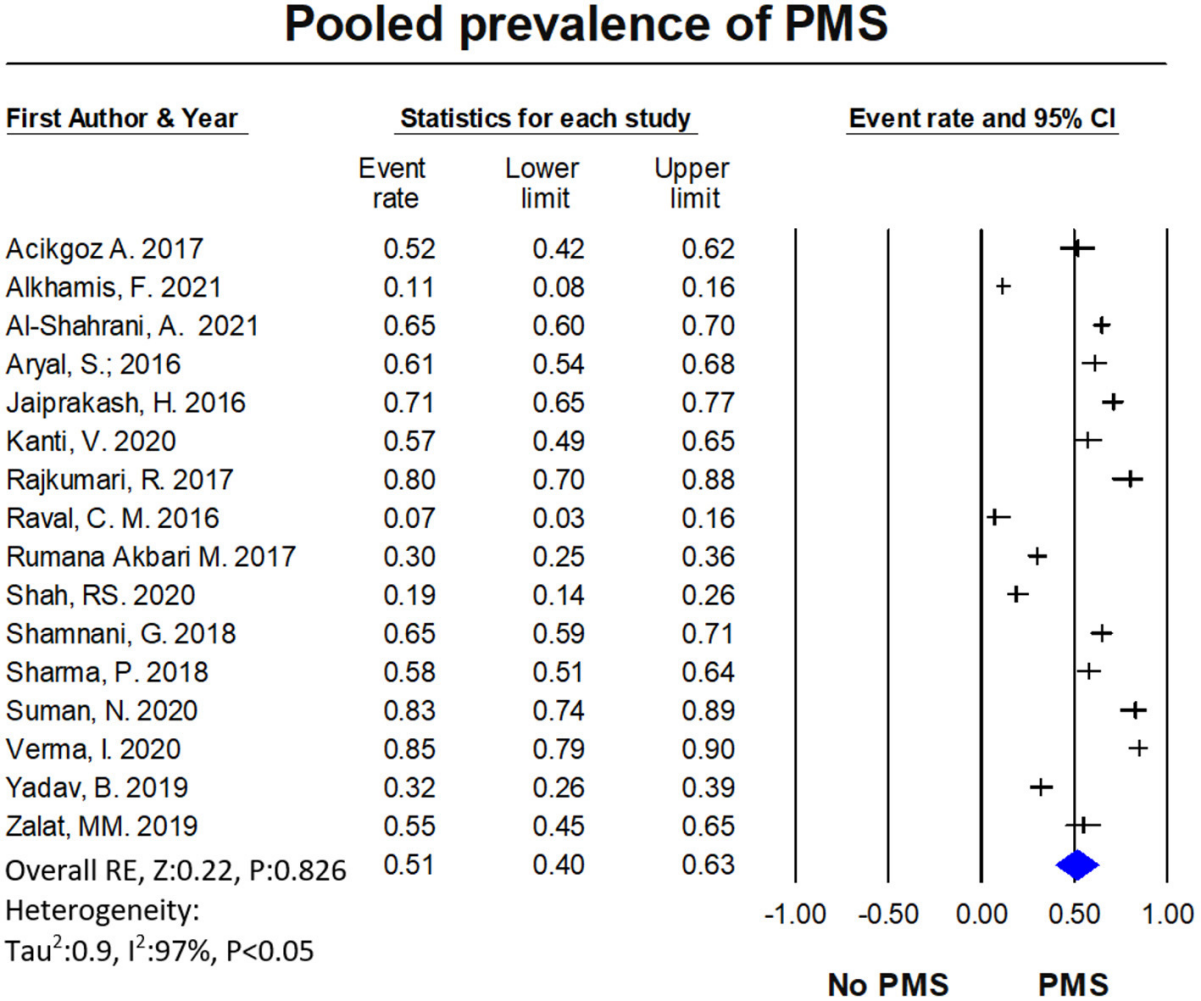
The forest plot of PMS prevalence. The diamond represents the overall results and 95% confidence interval of the random effect of the meta-analysis. Model: Random, overall effect size: 0.513, 95%CI: [0.396–0.629] (*z*-value: 0.220, *p*-value: 0.826), *I*^2^:96.93. Tau^2^: 0.901, s.e: 0.384, variance: 0.147, tau: 0.949.

**Figure 4:**
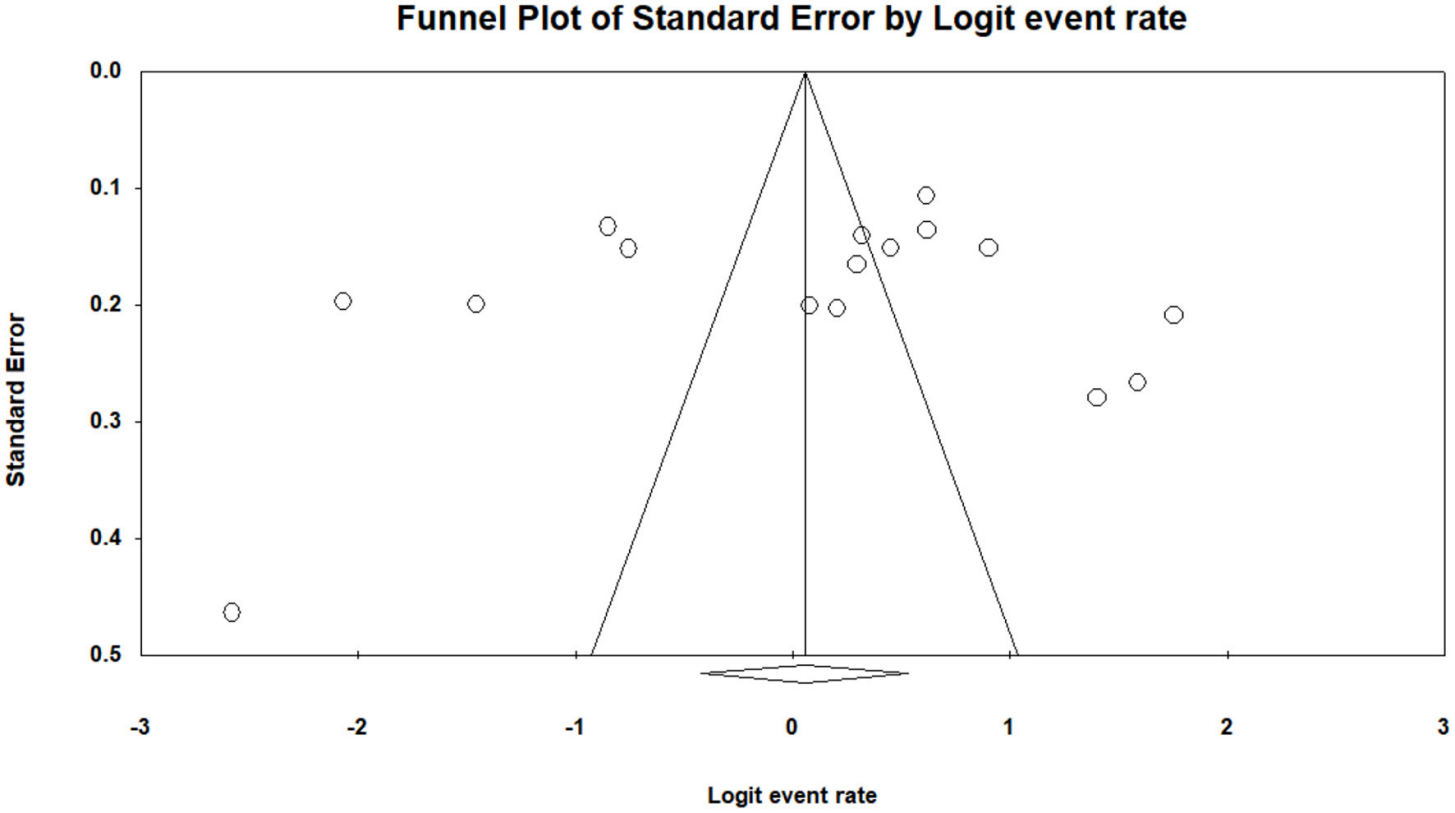
The funnel plot of pooled prevalence of PMS is asymmetry and suggest that overall effect of the analysis is biased. In this case the intercept (B0) is 1.06576, 95% confidence interval (−7.59403, 9.72556), with *t* = 0.27088, df = 11. The 1-tailed *p*-value (recommended) is 0.39575, and the 2-tailed *p*-value is 0.79150.

Among the total female medical students of the included studies (*n* = 4,874) across the continents, the prevalence of PMS was reported by 16 studies (*n* = 3,192). The pooled prevalence of PMS was 51.30% (95% CI: 0.396–0.629). The studies of PMS prevalence have been conducted mostly in Asia, Middle East and partly from European countries. Table 1 of the extracted data shows the country wise distribution of menstrual disturbances. Figure 3 shows the overall PMS prevalence with a high level of heterogeneity. Supplementary Figure 1 represents the pooled prevalence of PMS after excluding one study to minimize the heterogeneity. The highest prevalence of PMS was found from New Delhi, India (2), estimated at 85.20% (95% CI: 0.792–0.897) and lowest was from Gujarat, India (56) at 7% (95% CI: 0.030–0.158). This observation was in line with previous studies (17). The publication bias is indicated through the visual inspection of the funnel plot (Figure 4).

### Prevalence of PMDD

**Figure 5:**
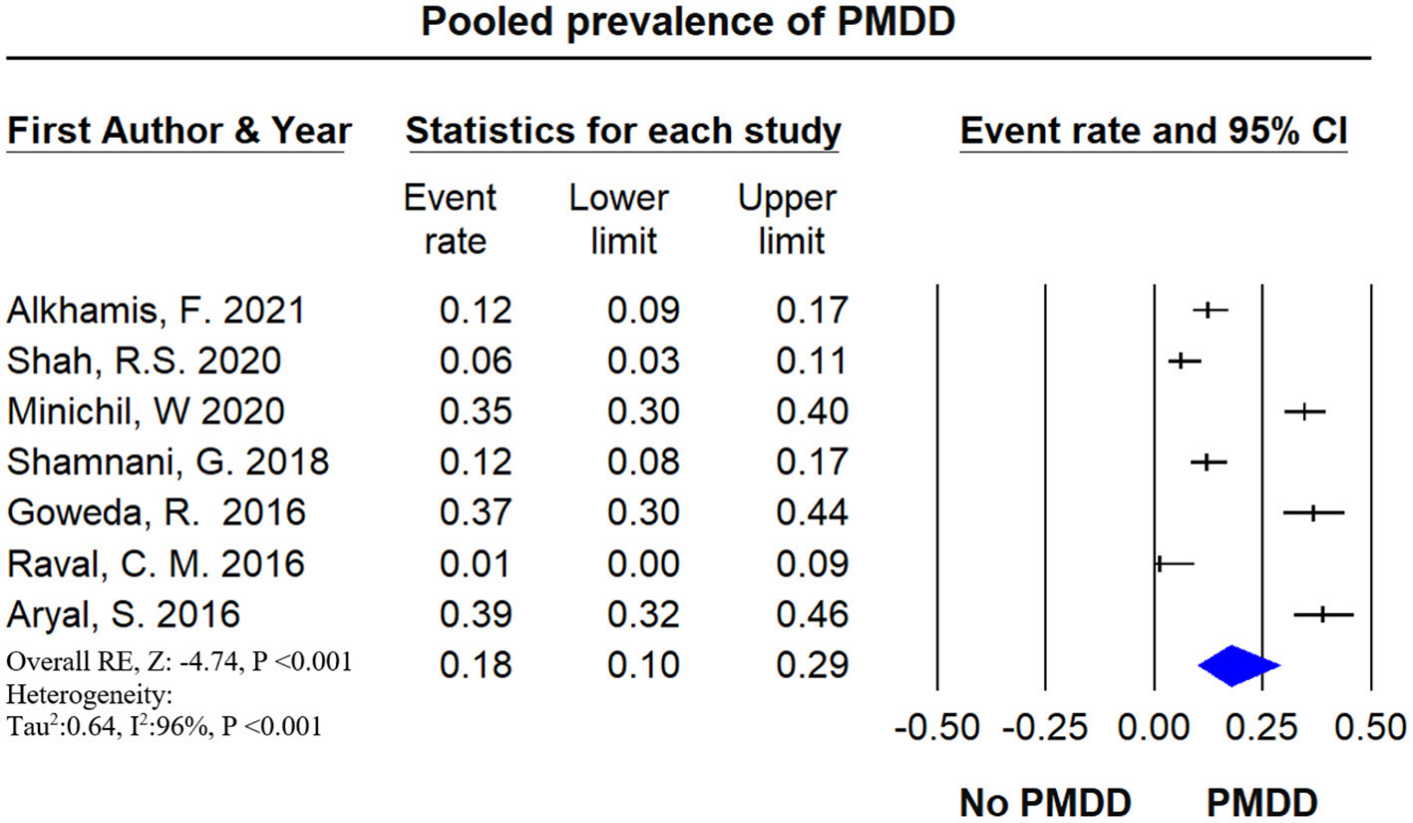
The forest plot of PMDD prevalence. The diamond represents the overall results and 95% confidence interval of the random effect of the meta-analysis. The prevalence of PMDD was reported in 6 studies including 1,487 participants. The estimated prevalence, pooled from all included studies for PMDD was found to be 17.7% (95% CI: 0.102–0.289) with a high level of heterogeneity (*I*^2^ = 95.12%), *p*-value: <0.001, Results: significant.

A total of 7 studies with a total of 1,487 female medical students were included in the meta-analysis for the prevalence of PMDD. Prevalence reported by individual studies ranged between 1 and 38%. The pooled estimate of PMDD was 17% (95% CI: 0.102–0.289). Figure 5 shows the overall PMDD prevalence. The heterogeneity test was not performed since the total number of PMDD studies were <10. Supplementary Figure 2 represents the pooled prevalence of PMS with exclusion of one study to minimize the heterogeneity.

### Prevalence of Dysmenorrhea

**Figure 6:**
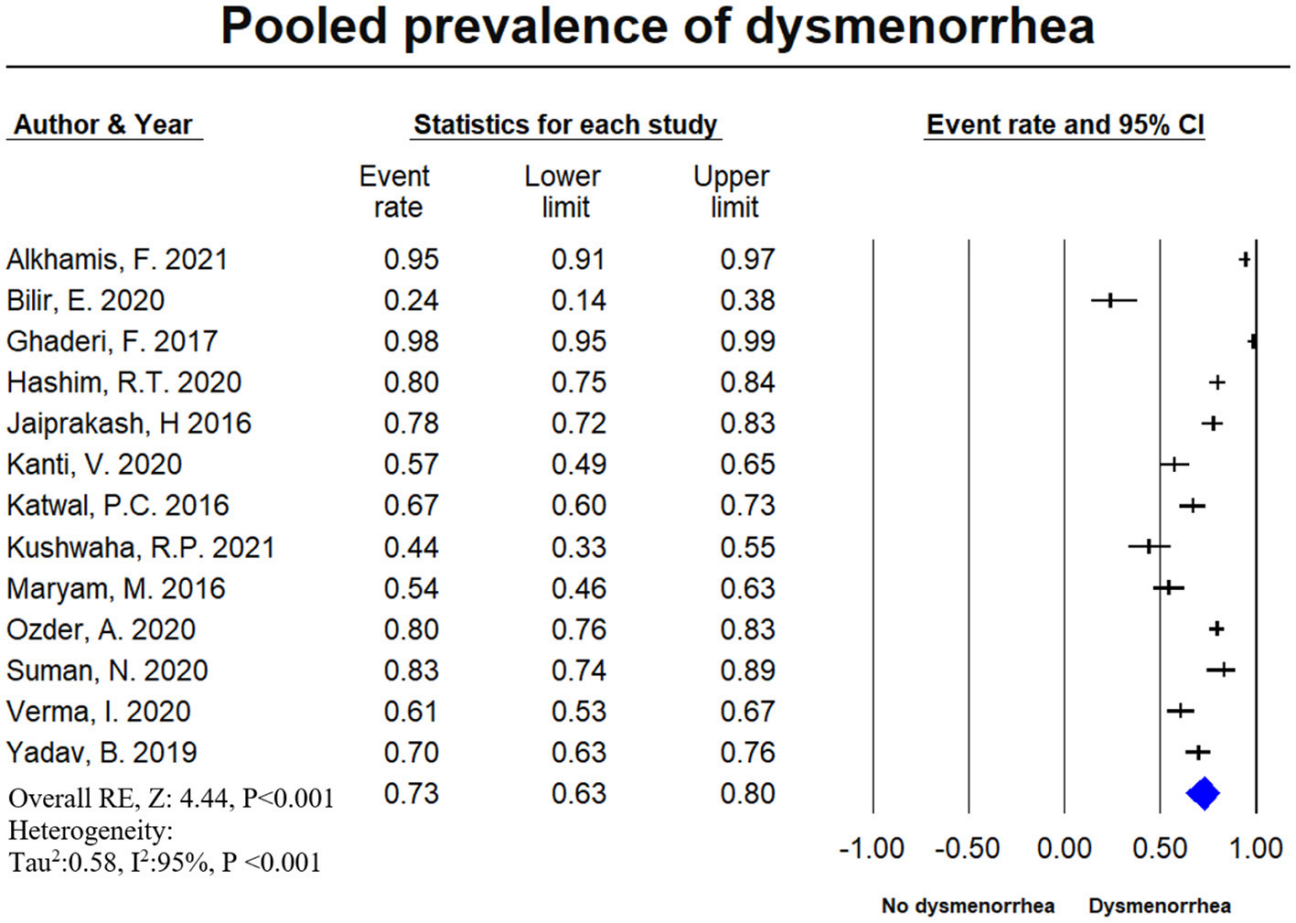
The forest plot of dysmenorrhea prevalence. The diamond represents the overall results and 95% confidence interval of the random effect of the meta-analysis. The prevalence of dysmenorrhea was reported in 13 studies including 2,497 participants. The estimated prevalence, pooled from all included studies for dysmenorrhea was found to be 17.7% (95% CI: 0.102–0.289) with a high level of heterogeneity (*I*^2^ = 94.7%), *p*-value: <0.001. Model name: Random, z value: 4.444, *p*-value <0.001, *I*^2^: 94.7%, tau^2^: 0.578, S.E: 0.283, variance: 0.08, Tau: 0.76.

**Figure 7:**
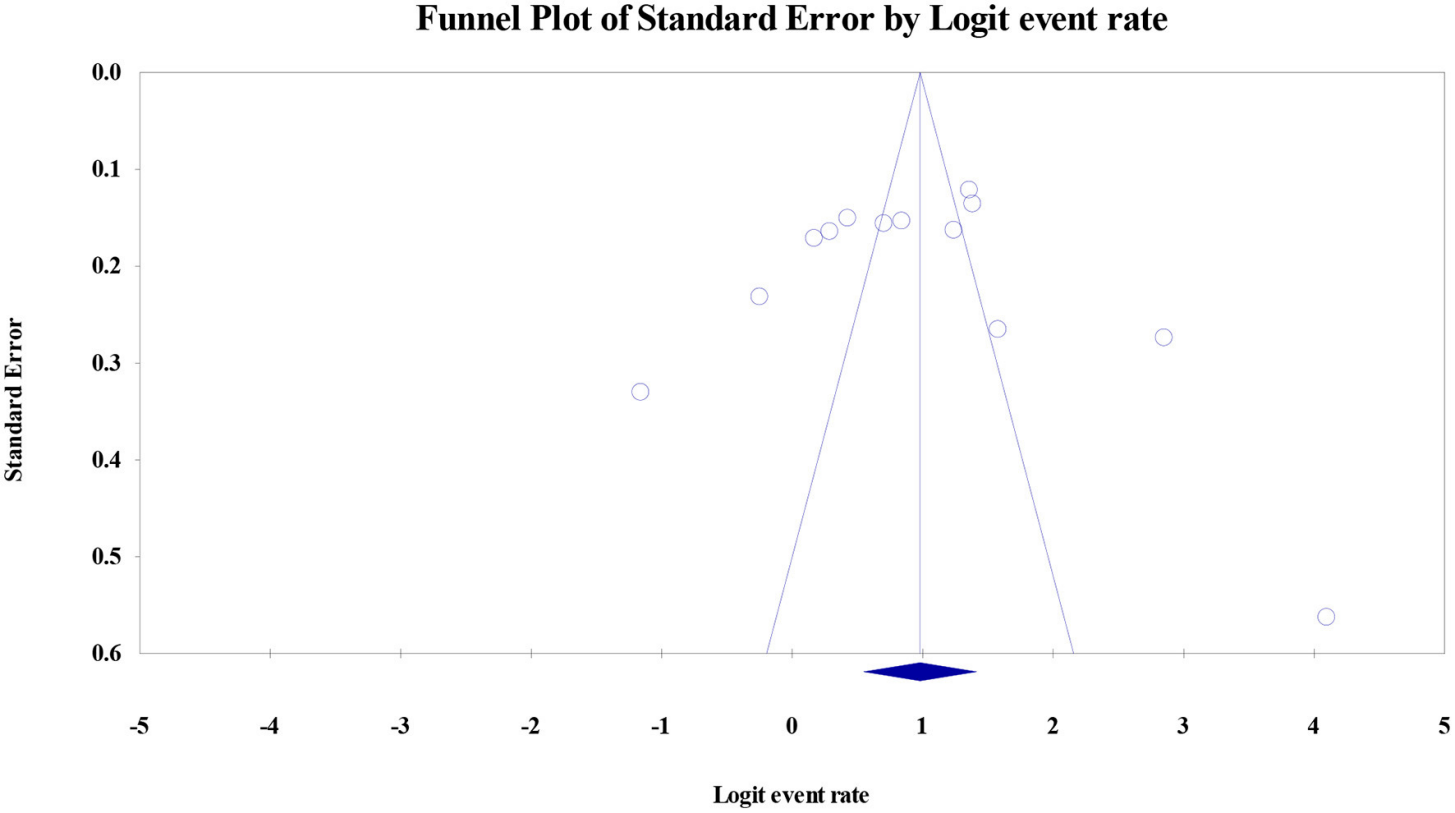
The funnel plot of pooled prevalence of dysmenorrhea is asymmetry and suggest that overall effect of the analysis is biased. In this case the intercept (B0) is 1.06576, 95% confidence interval (−7.59403, 9.72556), with *t* = 0.27088, df = 11. The 1-tailed *p*-value (recommended) is 0.39575, and the 2-tailed *p*-value is 0.79150.

A total of 13 studies with a total of 2,497 female medical students, were included in the meta-analysis for the prevalence of dysmenorrhea. Prevalence reported by individual studies ranged between 24 and 98%. The pooled estimate of dysmenorrhea was 72.7% (95% CI: 0.634–0.804) with a high level of heterogeneity (*I*^2^ = 94.7%). Figure 6 shows the overall dysmenorrhea prevalence. Supplementary Figure 3 represents the pooled prevalence of dysmenorrhea with exclusion of one study to minimize the heterogeneity. The publication bias is indicated through the visual inspection of the funnel plot (Figure 7) asymmetry.

## Discussion

This study is a first-of-its-kind prevalence study in medical students where a systematic review and meta-analysis of included studies were provided clearly. We searched the databases comprehensively and extracted the relevant data on menstrual disturbances in female medical students worldwide.

Although all humans experience some hormonal surges at puberty, women are predominantly susceptible to physical and mental stress caused by endocrinal changes associated with the menstrual cycle. Throughout the active reproductive life of a female (from menarche→regular menstrual cycle→pregnancy→menopause), the body goes through hormonal fluctuations, leading to stress and mood alterations. A high level of stress and mood disturbances can further complicate the menstrual cycle. The hypothalamic-pituitary-gonadal/adrenal (HPG/HPA) axis provides essential feedback and regulatory influence to maintain physiological homeostasis. Therefore, abnormality of either HPG or HPA is a major contributing factor for menstrual disturbances (58). Emotional stress could modulate these powerful feedback mechanisms by the HPA and HPG axis. For example, overactivation of HPA because of psychological stressors increases cortisol's plasma level to meet the increased energy demand in stressful situations (59, 60). Eventually, the higher cortisol concentration and the associated increase in metabolism disrupts the typical cyclical pattern of hormonal fluctuation leading to menstrual abnormalities such as PMS, PMDD, and dysmenorrhea.

### Evidence, Impact and Interventions

Pre-menstrual symptoms include a spectrum of psychosomatic symptoms arising in the luteal phase of the menstrual cycle affecting the typical lifestyle of a female and getting resolved after the menstruation cycle begins. The American Congress of Obstetricians and Gynecologists (ACOG) and the American Psychiatric Association (APA) both have a set of diagnostic criteria based on physical and psychological symptoms for diagnosing PMS and PMDD, respectively (7, 8). The physical and psychological symptoms generally occur between menarche and menopause anytime throughout the reproductive life. Pain during menstruation (period pain), known as dysmenorrhea, is widespread in young women. Dysmenorrhea affects approximately three-quarters of all women in their teens and early adult life. Women with menstrual disturbances have higher rates of negative impact on academic performance, work absences, higher medical expenses, and overall, lower quality of life (61).

We searched the databases comprehensively and extracted the relevant data on menstrual disturbances in female medical students worldwide. Our study found common complaints of PMS, PMDD, and dysmenorrhea in female medical students. Some studies have also reported a combination of menstrual disturbances (3, 5, 54). The calculated pooled prevalence of PMS was 51.30% (95% CI: 0.396–0.629), PMDD was 17.7% (95% CI: 0.102–0.289), and dysmenorrhea was 72.7% (95% CI: 0.102–0.289), respectively. The highest report of PMS was from New Delhi, India (2), and the lowest was from Gujarat, India (56). The highest report of PMDD was from Nepal (25), and the lowest was from Gujarat, India (56). The highest report of dysmenorrhea was from Iran (50), and the lowest was from Turkey (43). Could it be due to the food habit and lifestyle patterns? It would be interesting to explore this avenue further for targeted therapeutic interventions.

Although we searched comprehensively, we did not find a suitable study from the USA, Canada, UK, Australia, and other western countries. However, studies are available on the impact of therapeutic interventions for treatment of PMS and PMDD (62, 63). Likewise, Armour et al. provide a systematic review and meta-analysis of the prevalence of dysmenorrhea among 21,573 young women worldwide but the study does not focus on medical students (61). Medical students across the globe have been found to have higher levels of perceived stress and emotional distress. The higher stress level is due inability of students to tackle a challenging medical curriculum with a higher course load along with frequency of exams (64). Comparative research data indeed shows a higher level of perceived stress in medical students than the general population and students from another academic field (65, 66). Researchers have assessed perceived stress in medical students by specific questionnaires such as the Perceived Medical School Stress (PMSS) (64, 67). Rationally, menstrual disturbances and the associated discomfort add to the already increased perceived stress in medical students.

An exciting aspect of this review was to investigate root causes of the menstrual disturbances in medical students and the impact on academic and social life. Although we did not quantify the causal statistical data, our study highlights behavioral and lifestyle factors such as food and drink preferences, lack of exercise, smoking, caffeine intake, etc., may have a strong association with menstrual disturbances. It is also interesting to notice a trend of traditional interventions such as over-the-counter pain killers, drinking hot tea, etc., to reduce the symptoms of menstrual disturbances in medical students. This study supports evidence from previous observations by Kushwaha et al., who reported a lower incidence (19 vs. 48%) of moderate to severe dysmenorrhea in students who had been exercising regularly vs. students who did not. Most respondents adopted home remedies to manage painful menstruation without medical consultation. In addition, 61% respondents used analgesic drugs out of which 51% used Mefenamic acid as the most common self-medicated drug. The more concerning fact is that 78% had used drugs once a day, but 68% had insufficient knowledge about drug dosage (21). Long-term misuse of pain medications may lead to an additional burden of disease among medical students.

In accordance with the present results, a previous study by Alkhamis et al., reported a 23.3% prevalence of PMS and PMDD. Fifty-six percent of medical student respondents in the study were not involved in any exercise, and 21% did not sleep for 6 h or more. Sixty-five percent reported consuming caffeine regularly, and 26% reported eating junk food more often than others. Although no precise statistical analysis was shown between lifestyle behaviors and the prevalence of PMS and PMDD, a national association is possible. It was also reported that female students left early from the class because of PMS. Consequentially, 29.1% of the female medical students indicated low-grade scoring, and 22.5% reported lower grades than males. Among students with PMS or PMDD, 71.3% used hot drinks such as coffee, 57.4% used traditional pain killers as a mode to reduce pain. Only 11.2% opted for medical treatment (3). Therefore, a proper health policy and intervention should be implemented by all medical universities worldwide to provide female medical students with equal opportunity as their peers.

These results corroborate the ideas of Al-Shahrani et al., who reported that 50% students who experienced PMS did not exercise and 25% had a family history of menstrual problems. The quality of life was also significantly impaired in medical students because of menstruation disorder. More than 60% of the respondents did not use any medication or medical consultation to reduce the discomfort during the menstrual cycle (27).

Overall, this research paper shows direct and significant impact of menstrual disorders on the academic and personal quality of life of female medical students. Moreover, the academic disadvantage may lead to an unequal platform for success compared to other medical students. Therefore, this gap should be considered for a critical intervention by the medical universities, all stakeholders and government agencies.

### Equity Among All: A Call for Action

One of the early studies in 1971 by Kantero and Widholm on over 5,000 female adolescents reported a 43% irregular menstrual cycle rate with 20% having irregular menses 5 years after menarche (68). Furthermore, studies on non-medical college and school students reported chief complaints of oligomenorrhea or amenorrhea (16, 69). These early studies generated a considerable clinical interest to explore further the variability of menstrual patterns and associated symptoms such as PMS, PMDD, and dysmenorrhea. Early studies of menstrual patterns in medical students, however, report conflicting results. A study examined physical discomfort during menses in medical students and found no evidence to support the hypothesis that a higher perceived stress level was associated with a change of a menstrual function (70). On the contrary, an Indian study on medical students reported a PMS (67%) and dysmenorrhea (33%) as the most distressing problems associated with menstruation (48). These results seem to be consistent with other research which found that 16% had a positive correlation of irregular menses and examination stress (69). As discussed previously, in most of these early studies, the academic and quality of life were impaired because of menstrual disturbances.

Medical education has evolved in recent times not just to follow the government guidelines but also to fulfill the motto of education for all. Education trends have debunked the myth that only students of science background can apply and get into medical school. Many institutions have additional pre-medical programs tailored for non-medical students who wish to learn and practice medicine. In addition, a significant number of medical students in the USA require additional accommodations due to learning disabilities. Providing equal opportunities to all the sections of the students is advantageous for society and a fulfilling experience for a medical school. Currently, the USA has several practicing doctors who completed their medical studies with learning disabilities like ADHD (71). Therefore, medical educators should recognize the gap in academic performance and social index in the female medical students also as compared to their male peers and provide additional accessibility as required. The need of the hour is to recognize and urgently implement a knowledge-based program with additional measures to provide equal platforms for both male and female students.

### Strengths and Limitations

The current study is the first systematic review and meta-analysis of three predominant menstruation disturbances among female medical students worldwide. Present study adds value to the existing body of literature on female reproductive and personal wellbeing. The review also provides an overview of the traditional lifestyle factors leading to menstruation disturbances among medical students. In addition, the review also sheds light on the impact of menstrual disturbances in the academic and personal life. The present systematic review and meta-analysis may have overlooked some studies that could have been included in our review despite comprehensive search strategy. The present study could not find enough relevant studies from USA, UK, and Australia. Since the gray literature of the thesis and another non-peer-reviewed article were excluded, a piece of valuable information may have been lost because of this. The subgroup analysis of PMS, PMDD, and dysmenorrhea, could have been more in-depth, but that would also be too much information for a busy reader. A significant limitation of our study is the presence of a considerable level of heterogeneity. This could be due to the diverse geographical population with different cultures and differences in methodologies of the study protocol. The heterogeneity of results and different outcomes posed a major problem and could be resolved in the future by introducing a core outcome set but we still have to work on that. Present study includes Supplementary Figures 1–3 with more refined versions of forest plots by eliminating the one data to reduce the heterogeneity. Finally, the impact of menstrual disturbances on education and quality of life was not analyzed because of inadequate data. Therefore, the report may be underestimating the prevalence of the impact of menstrual disturbances.

## Conclusions

The current systematic review and meta-analysis shows a high prevalence of PMS and dysmenorrhea among female medical students. However, the prevalence of PMDD is relatively low in the same population. Most reproductive age group female students have impairment of academic and social quality of life because of menstrual symptoms. Lifestyle, socio-demographic, genetic, and psychological factors may contribute to the menstrual disturbances. Since there are a smaller number of studies and sharp variation of estimates in worldwide research data, it would be interesting in future to compare the prevalence of menstrual disturbances and associated factors between the female medical students of USA, UK, and Australia to the rest of the world in a large-scale study to find out the personal, professional, and economic impact of menstrual disturbances. Since a significant population of the studies either miss classes or have impairment of the academic performance, all stakeholders should step up the formulation of guidelines to increase our medical student population's overall quality of life. Early intervention in the medical universities and formulation of strategies to improve pain, mood, depression, and associated symptoms of menstrual disturbances would increase educational opportunities and foster a better quality of life. Finally, further studies are needed to evaluate and assess the cause of the menstrual disturbances and therapeutic interventions to bridge the gender gap in our society for a better tomorrow.

## Data Availability Statement

The original contributions presented in the study are included in the article/Supplementary Material, further inquiries can be directed to the corresponding author.

## Author Contributions

SM and SaN: conceptualizing, literature search and screening, methodology selection, data extraction, project administration, supervising and writing, and manuscript preparation. TC, JW, SaN, and RN: conceptualizing, data extraction and analysis, validation, writing, and reviewing and editing. RS: meta-analysis validation and data curation. RW and PR: conceptualizing, supervision, interpretation, and review and editing. RN, ShN, and SaN: final review and editing, data visualization, and project administration. All authors contributed to the article and approved the submitted version.

## Conflict of Interest

The authors declare that the research was conducted in the absence of any commercial or financial relationships that could be construed as a potential conflict of interest.

## Publisher's Note

All claims expressed in this article are solely those of the authors and do not necessarily represent those of their affiliated organizations, or those of the publisher, the editors and the reviewers. Any product that may be evaluated in this article, or claim that may be made by its manufacturer, is not guaranteed or endorsed by the publisher.

## References

[1] Hennegan J Winkler IT Bobel C Keiser D Hampton J Larsson G . Menstrual health: a definition for policy, practice, and research. Sex Reprod Health Matters. (2021) 29:1911618. 10.1080/26410397.2021.1911618 33910492 PMC8098749

[2] Verma I Joshi G Sood D Soni RK. Menstrual problems in undergraduate medical students: a cross-sectional study in a medical college of North India. J South Asian Federation Obstetr Gynaecol. (2020) 12:85–90. 10.5005/jp-journals-10006-1774

[3] Alkhamis F Almzraq LAA Alshayeb ZK AL-Jaziri ZY. Prevalence of premenstrual syndrome among medical students in King Faisal University in Alahssa-Saudi Arabia. Med Sci. (2021) 25:1971–83. Available online at: http://www.discoveryjournals.org/medicalscience/current_issue/v25/n114/A23.pdf21120003

[4] Hashim RT Alkhalifah SS Alsalman AA Alfaris DM Alhussaini MA Qasim RS . Prevalence of primary dysmenorrhea and its effect on the quality of life amongst female medical students at King Saud University, Riyadh, Saudi Arabia. Saudi Med J. (2020) 41:283–9. 10.15537/smj.2020.3.24988 32114601 PMC7841556

[5] Shah R Christian D. Association of socio-demographic, dietary and lifestyle factors with Premenstrual Syndrome (PMS) among undergraduate medical students of a tertiary care institute in Ahmedabad, Gujarat. J Family Med Prim Care. (2020) 9:5719–24. 10.4103/jfmpc.jfmpc_1553_20 33532420 PMC7842494

[6] Dilbaz B Aksan A. Premenstrual syndrome, a common but underrated entity: review of the clinical literature. J Turk German Gynecol Assoc. (2021) 22:139–48. 10.4274/jtgga.galenos.2021.2020.0133 33663193 PMC8187976

[7] American College of Obstetricians and Gynecologists. Guidelines for Women's Health Care: A Resource Manual. 4th ed. Washington, DC: The American College of Obstetricians and Gynecologists, Women's Health Care Physicians (2014). p. 889.

[8] Vahia V. Diagnostic and statistical manual of mental disorders 5: a quick glance. Indian *J Psychiatry*. (2013) 55:20–3. 10.4103/0019-5545.117131 24082241 PMC3777342

[9] Minichil W Eskindir E Demilew D Mirkena Y. Magnitude of premenstrual dysphoric disorder and its correlation with academic performance among female medical and health science students at University of Gondar, Ethiopia, 2019: a cross-sectional study. BMJ Open. (2020) 10:e034166. 10.1136/bmjopen-2019-034166 32727736 PMC7395298

[10] Duko B Mekuriaw B Molla A Ayano G. The prevalence of premenstrual dysphoric disorder among adolescents in Ethiopia: a systematic review and meta-analysis. Irish J Med Sci. (2020) 190:419–27. 10.1007/s11845-020-02275-7 32506277

[11] Osborn E Wittkowski A Brooks J Briggs PE O'Brien PMS. Women's experiences of receiving a diagnosis of premenstrual dysphoric disorder: a qualitative investigation. BMC Womens Health. (2020) 20:242. 10.1186/s12905-020-01100-8 33115437 PMC7594422

[12] Alshahrani MS. Dysmenorrhea and its effects among female students at health colleges in Najran University, Saudi Arabia: a cross-sectional study. J Women's Health Care. (2020) 9:484. 10.35248/2167-0420.20.9.484

[13] Karlsson TS Marions LB Edlund MG. Heavy menstrual bleeding significantly affects quality of life. Acta Obstetric Gynecol Scand. (2014) 93:52–7. 10.1111/aogs.12292 24266506

[14] Babbar K Martin J Ruiz J Parray AA Sommer M. Menstrual health is a public health and human rights issue. Lancet Public Health. (2021). 7:e10–e11. 10.1016/S2468-2667(21)00212-7 34717798 PMC8552814

[15] Bayeh E. The role of empowering women and achieving gender equality to the sustainable development of Ethiopia. Pac Sci Rev B Hum Soc Sci. (2016) 2:37–42. 10.1016/j.psrb.2016.09.013

[16] Munro AK Hunter EC Hossain SZ Keep M. A systematic review of the menstrual experiences of University students and the impacts on their education: a global perspective. PLoS ONE. (2021) 16:e0257333. 10.1371/journal.pone.0257333 34506544 PMC8432759

[17] Dutta A Sharma A. Prevalence of premenstrual syndrome and premenstrual dysphoric disorder in India: a systematic review and meta-analysis. Health Prom Perspect. (2021) 11:161–70. 10.34172/hpp.2021.20 34195039 PMC8233671

[18] Hashimoto K Fukushima K Fukushima N Sato H Yokota J Uchida K. Association between nutritional level, menstrual-related symptoms, and mental health in female medical students. Plos One (2020) 15(7). 10.1371/journal.pone.0235909 32658906 PMC7357753

[19] Strine TW Chapman DP Ahluwalia IB. Menstrual-related problems and psychological distress among women in the United States. J Womens Health. (2005) 14:316–23. 10.1089/jwh.2005.14.316 15916505

[20] Sahin N Kasap B Kirli U Yeniceri N Topal Y. Assessment of anxiety-depression levels and perceptions of quality of life in adolescents with dysmenorrhea. Reprod Health. (2018) 15:13. 10.1186/s12978-018-0453-3 29373981 PMC5787268

[21] Sah B Yadav P Sitaula S Sinha P Rai DS Sarraf DP . Evaluation of the severity and self-management practice in primary dysmenorrhea in medical and dental students: A cross-sectional study in a teaching hospital. Asian J Med Sci. (2021) 12:59–65. 10.3126/ajms.v12i3.32687

[22] Yesuf TA Eshete NA Sisay EA. Dysmenorrhea among University health science students, Northern Ethiopia: impact and associated factors. Int J Reprod Med. (2018) 2018:9730328. 10.1155/2018/9730328 29610764 PMC5828460

[23] Rafique N Al-Sheikh MH. Prevalence of menstrual problems and their association with psychological stress in young female students studying health sciences. Saudi Med J. (2018) 39:67–73. 10.15537/smj.2018.1.21438 29332111 PMC5885123

[24] Shamnani G Gupta V Jiwane R Singh S Tiwari S Bhartiy S. Prevalence of premenstrual syndrome and premenstrual dysphoric disorder among medical students and its impact on their academic and social performance. Natl J Physiol Pharm Pharmacol. (2018) 8:1205–8. 10.5455/njppp.2018.8.041572804201832727736

[25] Aryal S Thapa B Pant SB. Premenstrual syndrome and premenstrual dysphoric disorder in medical and nursing students of a tertiary care teaching hospital in Nepal. J Obstetr Gynaecol. (2017) 12:12–6. 10.3126/njog.v12i1.18975

[26] Lee LK Chen PC Lee KK Kaur J. Menstruation among adolescent girls in Malaysia: a cross-sectional school survey. Singapore Med J. (2006) 47:869–74. Available online at: http://www.sma.org.sg/smj/4710/4710a6.pdf 16990962

[27] Al-Shahrani AM Miskeen E Shroff F Elnour S Algahtani R Youssry I . Premenstrual syndrome and its impact on the quality of life of female medical students at Bisha University, Saudi Arabia. J Multidiscipl Healthc. (2021) 14:2373–9. 10.2147/JMDH.S327893 34475764 PMC8407666

[28] Farrokh-Eslamlou H Oshnouei S Heshmatian B Akbari E. Premenstrual syndrome and quality of life in Iranian medical students. Sex Reprod Healthc. (2015) 6:23–7. 10.1016/j.srhc.2014.06.009 25637421

[29] Salk RH Hyde JS Abramson LY. Gender differences in depression in representative national samples: META-analyses of diagnoses and symptoms. Psychol Bull. (2017) 143:783–822. 10.1037/bul0000102 28447828 PMC5532074

[30] Rotenstein LS Ramos MA Torre M Segal JB Peluso MJ Guille C . Prevalence of depression, depressive symptoms, and suicidal ideation among medical students. JAMA. (2016) 316:2214–36. 10.1001/jama.2016.17324 27923088 PMC5613659

[31] Dyrbye LN Thomas MR Shanafelt TD. Systematic review of depression, anxiety, and other indicators of psychological distress among U.S. and Canadian medical students. Acad Med. (2006) 81:354–73. 10.1097/00001888-200604000-00009 16565188

[32] Mirza AA Baig M Beyari GM Halawani MA Mirza AA. Depression and anxiety among medical students: a brief overview. Adv Med Educ Pract. (2021) 12:393–8. 10.2147/AMEP.S302897 33911913 PMC8071692

[33] Neufeld A Malin G. How medical students cope with stress: a cross-sectional look at strategies and their sociodemographic antecedents. BMC Med Educ. (2021) 21:299. 10.1186/s12909-021-02734-4 34034732 PMC8152145

[34] Majeed-Saidan MMA AlKharraz N Kaaki K AlTawil N Alenezy S Ahamed SS. Prevalence of premenstrual syndrome levels and its management among female students of medical and non-medical colleges in Riyadh. Cureus. (2020) 12:e11595. 10.7759/cureus.11595 33240729 PMC7681758

[35] Erbil N Geta TG Woldeamanuel GG Dassa TT. Prevalence and associated factors of premenstrual syndrome among women of the reproductive age group in Ethiopia: systematic review and meta-analysis. PLoS ONE. (2020) 15:e0241702. 10.1371/journal.pone.0241702 33156860 PMC7647055

[36] Lempp H Seale C. Medical students' perceptions in relation to ethnicity and gender: a qualitative study. BMC Med Educ. (2006) 6:17. 10.1186/1472-6920-6-17 16524457 PMC1435754

[37] Blanch DC Hall JA Roter DL Frankel RM. Medical student gender and issues of confidence. Patient Educ Couns. (2008) 72:374–81. 10.1016/j.pec.2008.05.021 18656322

[38] Ku MC. When does gender matter? Work Occup. (2011) 38:221–62. 10.1177/0730888410392319

[39] Stroup DF. Meta-analysis of observational studies in epidemiology. A proposal for reporting. JAMA. (2000) 283:2008–12. 10.1001/jama.283.15.2008 10789670

[40] Moher D. Preferred reporting items for systematic reviews and meta-analyses: the PRISMA statement. Ann Intern Med. (2009) 151:264–9, W64. 10.7326/0003-4819-151-4-200908180-00135 19622511

[41] Luchini C Stubbs B Solmi M Veronese N. Assessing the quality of studies in meta-analyses: advantages and limitations of the Newcastle Ottawa Scale. World J Meta Analy. (2017) 5:80–4. 10.13105/wjma.v5.i4.80

[42] Kanti V Verma V Singh NP. Study of menstrual abnormalities and its association with demographic factors among female medical students. J Clin Diagn Res. (2020). 14:QC06–QC09. 10.7860/JCDR/2020/44086.13957

[43] Bilir E Yildiz S Yakin K Ata B. The impact of dysmenorrhea and premenstrual syndrome on academic performance of college students, and their willingness to seek help. J Turk Soc Obstetr Gynecol. (2020) 17:196–201. 10.4274/tjod.galenos.2020.97266 33072424 PMC7538819

[44] Özder A Salduz Z. The prevalence of dysmenorrhea and its effects on female University students- quality of life: what can we do in primary care? Int J Clin Exp Med. (2020) 13:6496–505. Avialable online at: https://avesis.bezmialem.edu.tr/yayin/0028d8c5-6383-4b2d-a032-fd3ec6ee6e5d/the-prevalence-of-dysmenorrhea-and-its-effects-on-female-university-students-quality-of-life-what-can-we-do-in-primary-care/document.pdf34749716

[45] Nama S Gaddala A Matli P Rajamouli J Gurnule S Tejaswi U. A Cross sectional study to assess the prevalence of menstrual abnormalities in medical students of Karimnagar. Eur J Mol Clin Med. (2021) 7:6959–65. Available online at: https://ejmcm.com/pdf_9633_480e2ed047eb0e0f57a2977672250686.html

[46] Zalat MM Azam AN Aahmadi HG Alhazmi NO Alshaya RA. Prevalence of premenstrual syndrome among Taibah University female medical students, KSA. Int J Health Med Curr Res. (2019) 4:1469–77. 10.22301/IJHMCR.2528-3189.1469

[47] Yadav B Taneja P. Questionnaire based study on menstrual patterns among female medical University students of rural North India. J Evol Med Dent Sci. (2019) 8:1232. 10.14260/jemds/2019/273

[48] Sharma P Patro A Ibrahim S Reddy TSK Jain N Mallya SD. Premenstrual symptoms and lifestyle Factors Associated with it among Medical students. Indian J Public Health Res. Dev. (2018) 9:39–45. 10.5958/0976-5506.2018.01312.827212819

[49] Rajkumari R Keithellakpam S Thiyam J Devi NM. Relationship between psychosocial stress and menstrual function-related abnormalities among the female undergraduate medical students. J Evol Med Dent Sci. (2017) 6:3103–7. 10.14260/Jemds/2017/669

[50] Ghaderi F Asghari Jafarabadi M Mohseni Bandpei MA. Dysmenorrhea and self-care strategies in Iranian female students: a regression modeling of pain severity and underlying factors. Int J Adolesc Med Health. (2017) 29:20160017. 10.1515/ijamh-2016-0017 27428842

[51] Acikgoz A Dayi A Binbay T. Prevalence of premenstrual syndrome and its relationship to depressive symptoms in first-year University students. Saudi Med J. (2017) 38:1125–31. 10.15537/smj.2017.11.20526 29114701 PMC5767616

[52] Rumana A Sudharani M Kallupurackal S Ramya V Nagendra G Suryakantha A. Prevalence of premenstrual syndrome among medical students. Natl J Community Med. (2017) 8:292–4. Availble online at: http://www.njcmindia.org/uploads/8-6_292-294.pdf33240729

[53] Katwal PC Karki NR Sharma P Tamrakar SR. Dysmenorrhea and stress among the nepalese medical students. Kathmandu Univ Med J. (2016) 14:318–21. 29336418

[54] Jaiprakash H Myint K Chai L Nasir B. Prevalence of dysmenorrhea and its sequel among medical students in a Malaysian University. Br J Med Med Res. (2016) 16:1–8. 10.9734/BJMMR/2016/25135

[55] Goweda RA Alkot MM Alturkistani FA Alhajaji RJ Aljebali SS Baashr ZA. Prevalence of premenstrual dysphoric disorder among medical students of Umm Al-Qura University, Makkah Al-Mukaramah, Kingdom of Saudi Arabia. Middle East J Family Med. (2016) 14:14–20. 10.5742/MEWFM.2015.92785

[56] Raval C Panchal B Tiwari D Vala A Bhatt R. Prevalence of premenstrual syndrome and premenstrual dysphoric disorder among college students of Bhavnagar, Gujarat. Indian J Psychiatry. (2016) 58:164–70. 10.4103/0019-5545.183796 27385849 PMC4919960

[57] Maryam Ritonga MA Istriati. Relationship between menstrual profile and psychological stress with dysmenorrhea. Althea Med J. (2016) 3:382–7. 10.15850/amj.v3n3.884

[58] Oyola MG Handa RJ. Hypothalamic–pituitary–adrenal and hypothalamic–pituitary–gonadal axes: sex differences in regulation of stress responsivity. Stress. (2017) 20:476–94. 10.1080/10253890.2017.1369523 28859530 PMC5815295

[59] Juruena MF. Early-life stress and HPA axis trigger recurrent adulthood depression. Epilepsy Behav. (2014) 38:148–59. 10.1016/j.yebeh.2013.10.020 24269030

[60] Herman JP McKlveen JM Ghosal S Kopp B Wulsin A Makinson R . Regulation of the hypothalamic-pituitary-adrenocortical stress response. Compreh Physiol. (2016) 6:603–21. 10.1002/cphy.c150015 27065163 PMC4867107

[61] Armour M Parry K Manohar N Holmes K Ferfolja T Curry C . The prevalence and academic impact of dysmenorrhea in 21,573 young women: a systematic review and meta-analysis. J Womens Health. (2019) 28:1161–71. 10.1089/jwh.2018.7615 31170024

[62] Kleinstauber M Witthoft M Hiller W. Cognitive-behavioral and pharmacological interventions for premenstrual syndrome or premenstrual dysphoric disorder: a meta-analysis. J Clin Psychol Med Settings. (2012) 19:308–19. 10.1007/s10880-012-9299-y 22426857

[63] Shah NR Jones JB Aperi J Shemtov R Karne A Borenstein J. Selective serotonin reuptake inhibitors for premenstrual syndrome and premenstrual dysphoric disorder: a meta-analysis. Obstet Gynecol. (2008) 111:1175–82. 10.1097/AOG.0b013e31816fd73b 18448752 PMC2670364

[64] Heinen I Bullinger M Kocalevent R-D. Perceived stress in first year medical students - associations with personal resources and emotional distress. BMC Med Educ. (2017) 17:4. 10.1186/s12909-016-0841-8 28056972 PMC5216588

[65] Dyrbye LN Harper W Durning SJ Moutier C Thomas MR Massie FS Jr . Patterns of distress in US medical students. Med Teach. (2011) 33:834–9. 10.3109/0142159X.2010.531158 21942482

[66] Dyrbye LN Shanafelt TD. Commentary: medical student distress: a call to action. Acad Med. (2011) 86:801–3. 10.1097/ACM.0b013e31821da481 21715992

[67] Fasoro AA Oluwadare T Ojo TF Oni IO. Perceived stress and stressors among first-year undergraduate students at a private medical school in Nigeria. J Taibah Univ Med Sci. (2019) 14:425–30. 10.1016/j.jtumed.2019.08.003 31728140 PMC6838949

[68] Widholm O Kantero R-L. III Menstrual patterns of adolescent girls according to chronological and gynecological ages. Acta Obstetric Gynecol Scand. (1971) 50:19–29. 10.3109/00016347109155077

[69] Demir SC Kadayýfçý TO Vardar MA Atay Y. Dysfunctional uterine bleeding and other menstrual problems of secondary school students in Adana, Turkey. J Pediatr Adolesc Gynecol. (2000) 13:171–5. 10.1016/S1083-3188(00)00061-9 11173019

[70] Clarvit SR. Stress and menstrual dysfunction in medical students. Psychosomatics. (1988) 29:404–9. 10.1016/S0033-3182(88)72341-5 3227095

[71] Zazove P Case B Moreland C Plegue MA Hoekstra A Ouellette A . U.S. medical schools' compliance with the americans with disabilities act: findings from a national study. Acad Med. (2016) 91:979–86. 10.1097/ACM.0000000000001087 26796093

