# Supplementary material for: Academic and Social Impact of Menstrual Disturbances in Female Medical Students: A Systematic Review and Meta-Analysis
Source: Front Med (Lausanne). 2022 Feb 15;9:821908. doi: 10.3389/fmed.2022.821908 (PMC8886240; doi:10.3389/fmed.2022.821908)
Supplement: Supplementary file 1 [file Data_Sheet_1.PDF]

Supplementary Material

Supplementary Figure 1- One study removed: Pooled prevalence of PMS

One study removed: Pooled prevalence of PMS

First Author & Year	Statistics with study removed: Point	Lower limit	Upper limit
Acikgoz A. 2017	0.51	0.39	0.64
Alkhamis, F. 2021	0.55	0.44	0.65
Al-Shahrani, A. 2021	0.50	0.38	0.63
Aryal, S.; 2016	0.51	0.38	0.63
Jaiprakash, H. 2016	0.50	0.38	0.62
Kanti, V. 2020	0.51	0.38	0.63
Rajkumari, R. 2017	0.49	0.37	0.61
Raval, C. M. 2016	0.55	0.43	0.66
Rumana Akbari M. 2017	0.53	0.41	0.65
Shah, RS. 2020	0.54	0.42	0.65
Shamnani, G. 2018	0.50	0.38	0.63
Sharma, P. 2018	0.51	0.38	0.63
Suman, N. 2020	0.49	0.37	0.61
Verma, I. 2020	0.49	0.37	0.60
Yadav, B. 2019	0.53	0.40	0.65
Zalat, MM. 2019	0.51	0.39	0.63

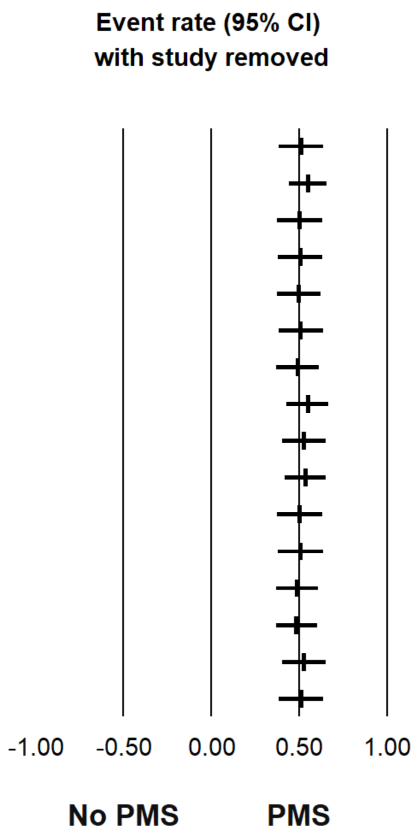

Supplementary Figure 2- One study removed: Pooled prevalence of PMDD

One study removed: Pooled prevalence of PMDD

First Author & Year	Statistics with study removed: Point	Lower limit	Upper limit
Alkhamis, F. 2021	0.19	0.11	0.31
Shah, R.S. 2020	0.21	0.13	0.33
Minichil, W 2020	0.15	0.07	0.28
Shamnani, G. 2018	0.19	0.11	0.31
Goweda, R. 2016	0.15	0.07	0.27
Raval, C. M. 2016	0.21	0.12	0.33
Aryal, S. 2016	0.15	0.07	0.27

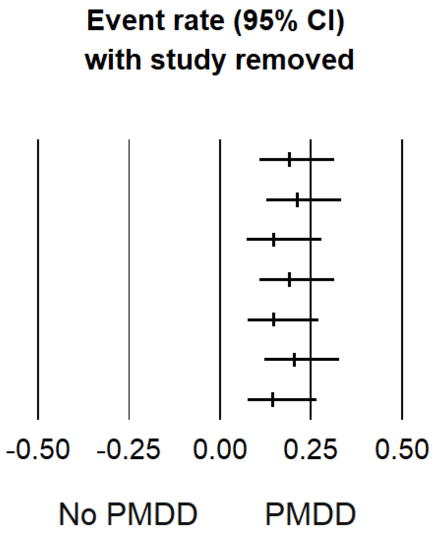

3 Supplementary Figure 3- One study removed: Pooled prevalence of dysmenorrhea

One study removed: Pooled prevalence of dysmenorrhea

Author & Year	Statistics with study removed: Point	Lower limit	Upper limit
Alkhamis, F. 2021	0.69	0.60	0.77
Bilir, E. 2020	0.76	0.67	0.82
Ghaderi, F. 2017	0.69	0.60	0.77
Hashim, R.T. 2020	0.72	0.62	0.80
Jaiprakash, H 2016	0.72	0.62	0.81
Kanti, V. 2020	0.74	0.64	0.82
Katwal, P.C. 2016	0.73	0.63	0.82
Kushwaha, R.P. 2021	0.75	0.66	0.82
Maryam, M. 2016	0.74	0.65	0.82
Ozder, A. 2020	0.72	0.62	0.81
Suman, N. 2020	0.72	0.62	0.80
Verma, I. 2020	0.74	0.64	0.82
Yadav, B. 2019	0.73	0.63	0.81

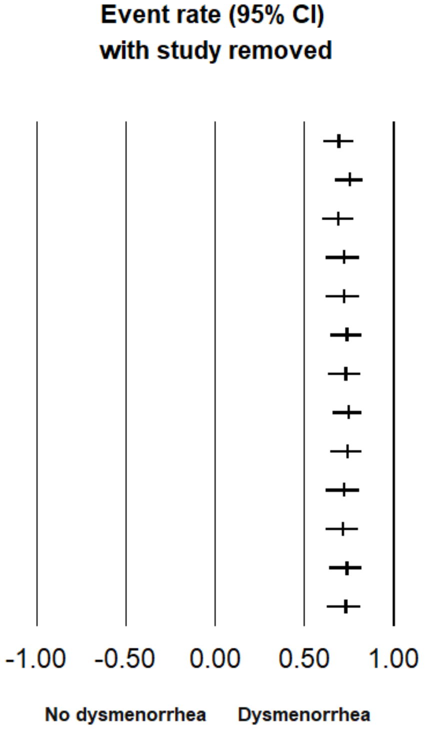

Supplementary Table 1- Modified NOS scale raw data

Author/Year	Sample representativeness	Sample size/Was the sample size adequate (Did the authors conducted a sample size calculation to determine an adequate sample size)	Response rate	Assessment of outcome	Statistical tests	Total Score
Alkhamis, F; 2021	a) truly representative of the average __________(describe) in the community*<br>b) somewhat representative of the average _____ in the community<br>c) selected group of users eg nurses, volunteers<br>d) no description of the derivation of the cohort	a) Justified and satisfactory.<br>b) Not justified.	a) Comparability between respondents and non-respondents characteristics is established, and the response rate is satisfactory.<br>b) The response rate is unsatisfactory, or the comparability between respondents and non-respondents is unsatisfactory.<br>c) No description of the response rate or the characteristics of the responders and the non-responders.	a) independent blind assessment<br>b) record linkage<br>c) self-report *<br>d) no description	a) The statistical test used to analyze the data is clearly described and appropriate, and the measurement of the association is presented, including confidence intervals and the probability level (p value). * b) The statistical test is not appropriate, not described or incomplete.	***
Ramayan Prasad Kushwaha; 2021	a) truly representative of the average __________(describe) in the community*<br>b) somewhat representative of the average _____ in the community<br>c) selected group of users eg nurses, volunteers<br>d) no description of the derivation of the cohort	a) Justified and satisfactory*<br>b) Not justified.	a) Comparability between respondents and non-respondents characteristics is established, and the response rate is satisfactory<br>b) The response rate is unsatisfactory, or the comparability between respondents and non-respondents is unsatisfactory<br>c) No description of the response rate or the characteristics of the responders and the non-responders	a) independent blind assessment<br>b) record linkage<br>c) self-report*<br>d) no description	a) The statistical test used to analyze the data is clearly described and appropriate, and the measurement of the association is presented, including confidence intervals and the probability level (p value).* b) The statistical test is not appropriate, not described or incomplete.	****
Al-Shahrani, A, 2021	a) truly representative of the average __________(describe) in the community *<br>b) somewhat representative of the average _____ in the community<br>c) selected group of users eg nurses, volunteers<br>d) no description of the derivation of the cohort	a) Justified and satisfactory.<br>b) Not justified.	a) Comparability between respondents and non-respondents characteristics is established, and the response rate is satisfactory.<br>b) The response rate is unsatisfactory, or the comparability between respondents and non-respondents is unsatisfactory.<br>c) No description of the response rate or the characteristics of the responders and the non-responders.	a) independent blind assessment<br>b) record linkage<br>c) self-report *<br>d) no description	a) The statistical test used to analyze the data is clearly described and appropriate, and the measurement of the association is presented, including confidence intervals and the probability level (p value). * b) The statistical test is not appropriate, not described or incomplete.	***
Majeed-Saidan,	a) truly representative of the average __________(describe)	a) Justified and satisfactory *<br>b) Not justified.	a) Comparability between respondents and non-respondents	a) independent blind	a) The statistical test used to analyze the data is clearly described and appropriate, and the	**

MMA; 2020	in the community<br>b) somewhat representative of the average _____ in the community<br>c) selected group of users eg nurses, volunteers<br>d) no description of the derivation of the cohort		characteristics is established, and the response rate is satisfactory.<br>b) The response rate is unsatisfactory, or the comparability between respondents and non-respondents is unsatisfactory.<br>c) No description of the response rate or the characteristics of the responders and the non-responders.	assessment<br>b) record linkage<br>c) self-report *<br>d) no description	measurement of the association is presented, including confidence intervals and the probability level (p value). b) The statistical test is not appropriate, not described or incomplete.	
Shah, RS; 2020	a) truly representative of the average __________(describe) in the community*<br>b) somewhat representative of the average _____ in the community<br>c) selected group of users eg nurses, volunteers<br>d) no description of the derivation of the cohort	a) Justified and satisfactory *<br>b) Not justified.	a) Comparability between respondents and non-respondents characteristics is established, and the response rate is satisfactory.<br>b) The response rate is unsatisfactory, or the comparability between respondents and non-respondents is unsatisfactory.<br>c) No description of the response rate or the characteristics of the responders and the non-responders.	a) independent blind assessment<br>b) record linkage<br>c) self-report *<br>d) no description	a) The statistical test used to analyze the data is clearly described and appropriate, and the measurement of the association is presented, including confidence intervals and the probability level (p value). * b) The statistical test is not appropriate, not described or incomplete.	****
Kanti, V; 2020	a) truly representative of the average __________(describe) in the community<br>b) somewhat representative of the average _____ in the community *<br>c) selected group of users eg nurses, volunteers<br>d) no description of the derivation of the cohort	a) Justified and satisfactory<br>b) Not justified.	a) Comparability between respondents and non-respondents characteristics is established, and the response rate is satisfactory.<br>b) The response rate is unsatisfactory, or the comparability between respondents and non-respondents is unsatisfactory.<br>c) No description of the response rate or the characteristics of the responders and the non-responders.	a) independent blind assessment<br>b) record linkage<br>c) self-report *<br>d) no description	a) The statistical test used to analyze the data is clearly described and appropriate, and the measurement of the association is presented, including confidence intervals and the probability level (p value). * b) The statistical test is not appropriate, not described or incomplete.	***
Minichil, W; 2020	a) truly representative of the average __________(describe) in the community*<br>b) somewhat representative of the average _____ in the community<br>c) selected group of users eg nurses, volunteers<br>d) no description of the derivation of the cohort	a) Justified and satisfactory*<br>b) Not justified.	a) Comparability between respondents and non-respondents characteristics is established, and the response rate is satisfactory.<br>b) The response rate is unsatisfactory, or the comparability between respondents and non-respondents is unsatisfactory.<br>c) No description of the response rate or the characteristics of the responders and the non-responders.	a) independent blind assessment<br>b) record linkage<br>c) self-report *<br>d) no description	a) The statistical test used to analyze the data is clearly described and appropriate, and the measurement of the association is presented, including confidence intervals and the probability level (p value). * b) The statistical test is not appropriate, not described or incomplete.	****

Bilir, E; 2020	a) truly representative of the average __________(describe) in the community*<br>b) somewhat representative of the average _____ in the community<br>c) selected group of users eg nurses, volunteers<br>d) no description of the derivation of the cohort	a) Justified and satisfactory*<br>b) Not justified.	a) Comparability between respondents and non-respondents characteristics is established, and the response rate is satisfactory*<br>b) The response rate is unsatisfactory, or the comparability between respondents and non-respondents is unsatisfactory.<br>c) No description of the response rate or the characteristics of the responders and the non-responders.	a) independent blind assessment<br>b) record linkage<br>c) self-report *<br>d) no description	a) The statistical test used to analyze the data is clearly described and appropriate, and the measurement of the association is presented, including confidence intervals and the probability level (p value). * b) The statistical test is not appropriate, not described or incomplete.	*****
Ozder, A; 2020	a) truly representative of the average __________(describe) in the community<br>b) somewhat representative of the average _____ in the community *<br>c) selected group of users eg nurses, volunteers<br>d) no description of the derivation of the cohort	a) Justified and satisfactory<br>b) Not justified.	a) Comparability between respondents and non-respondents characteristics is established, and the response rate is satisfactory<br>b) The response rate is unsatisfactory, or the comparability between respondents and non-respondents is unsatisfactory.<br>c) No description of the response rate or the characteristics of the responders and the non-responders.	a) independent blind assessment<br>b) record linkage<br>c) self-report *<br>d) no description	a) The statistical test used to analyze the data is clearly described and appropriate, and the measurement of the association is presented, including confidence intervals and the probability level (p value). * b) The statistical test is not appropriate, not described or incomplete.	***
Verma Indu; 2020	a) truly representative of the average __________(describe) in the community<br>b) somewhat representative of the average _____ in the community *<br>c) selected group of users eg nurses, volunteers<br>d) no description of the derivation of the cohort	a) Justified and satisfactory<br>b) Not justified.	a) Comparability between respondents and non-respondents characteristics is established, and the response rate is satisfactory<br>b) The response rate is unsatisfactory, or the comparability between respondents and non-respondents is unsatisfactory.<br>c) No description of the response rate or the characteristics of the responders and the non-responders.	a) independent blind assessment<br>b) record linkage<br>c) self-report *<br>d) no description	a) The statistical test used to analyze the data is clearly described and appropriate, and the measurement of the association is presented, including confidence intervals and the probability level (p value). * b) The statistical test is not appropriate, not described or incomplete.	***
Hashim, R.T.; 2021	a) truly representative of the average __________(describe) in the community *<br>b) somewhat representative of the average _____ in the community<br>c) selected group of users eg nurses, volunteers<br>d) no description of the derivation of the cohort	a) Justified and satisfactory *<br>b) Not justified.	a) Comparability between respondents and non-respondents characteristics is established, and the response rate is satisfactory*<br>b) The response rate is unsatisfactory, or the comparability between respondents and non-respondents is unsatisfactory.<br>c) No description of the response	a) independent blind assessment<br>b) record linkage<br>c) self-report *<br>d) no description	a) The statistical test used to analyze the data is clearly described and appropriate, and the measurement of the association is presented, including confidence intervals and the probability level (p value). * b) The statistical test is not appropriate, not described or incomplete.	*****

			rate or the characteristics of the responders and the non-responders.			
Suman Nama, 2020	a) truly representative of the average __________(describe) in the community<br>b) somewhat representative of the average _____ in the community *<br>c) selected group of users eg nurses, volunteers<br>d) no description of the derivation of the cohort	a) Justified and satisfactory<br>b) Not justified.	a) Comparability between respondents and non-respondents characteristics is established, and the response rate is satisfactory<br>b) The response rate is unsatisfactory, or the comparability between respondents and non-respondents is unsatisfactory.<br>c) No description of the response rate or the characteristics of the responders and the non-responders.	a) independent blind assessment<br>b) record linkage<br>c) self-report *<br>d) no description	a) The statistical test used to analyze the data is clearly described and appropriate, and the measurement of the association is presented, including confidence intervals and the probability level (p value). * b) The statistical test is not appropriate, not described or incomplete.	***
Marwa Mohamed Zalat, 2019	a) truly representative of the average __________(describe) in the community<br>b) somewhat representative of the average _____ in the community *<br>c) selected group of users eg nurses, volunteers<br>d) no description of the derivation of the cohort	a) Justified and satisfactory<br>b) Not justified.	a) Comparability between respondents and non-respondents characteristics is established, and the response rate is satisfactory<br>b) The response rate is unsatisfactory, or the comparability between respondents and non-respondents is unsatisfactory.<br>c) No description of the response rate or the characteristics of the responders and the non-responders.	a) independent blind assessment<br>b) record linkage<br>c) self-report *<br>d) no description	a) The statistical test used to analyze the data is clearly described and appropriate, and the measurement of the association is presented, including confidence intervals and the probability level (p value). * b) The statistical test is not appropriate, not described or incomplete.	***
Yadav, B; 2019	a) truly representative of the average __________(describe) in the community<br>b) somewhat representative of the average _____ in the community *<br>c) selected group of users eg nurses, volunteers<br>d) no description of the derivation of the cohort	a) Justified and satisfactory<br>b) Not justified.	a) Comparability between respondents and non-respondents characteristics is established, and the response rate is satisfactory<br>b) The response rate is unsatisfactory, or the comparability between respondents and non-respondents is unsatisfactory.<br>c) No description of the response rate or the characteristics of the responders and the non-responders.	a) independent blind assessment<br>b) record linkage<br>c) self-report *<br>d) no description	a) The statistical test used to analyze the data is clearly described and appropriate, and the measurement of the association is presented, including confidence intervals and the probability level (p value). * b) The statistical test is not appropriate, not described or incomplete.	***
Sharma, P.; 2018	a) truly representative of the average __________(describe) in the community*<br>b) somewhat representative of the average _____ in the community<br>c) selected group of users eg nurses, volunteers	a) Justified and satisfactory*<br>b) Not justified.	a) Comparability between respondents and non-respondents characteristics is established, and the response rate is satisfactory<br>b) The response rate is unsatisfactory, or the comparability between respondents and non-respondents	a) independent blind assessment<br>b) record linkage<br>c) self-report *<br>d) no description	a) The statistical test used to analyze the data is clearly described and appropriate, and the measurement of the association is presented, including confidence intervals and the probability level (p value). * b) The statistical test is not appropriate, not described or incomplete.	****

	d) no description of the derivation of the cohort		is unsatisfactory.<br>c) No description of the response rate or the characteristics of the responders and the non-responders.			
Shamnani, G.; 2018	a) truly representative of the average __________(describe) in the community<br>b) somewhat representative of the average _____ in the community *<br>c) selected group of users eg nurses, volunteers<br>d) no description of the derivation of the cohort	a) Justified and satisfactory<br>b) Not justified.	a) Comparability between respondents and non-respondents characteristics is established, and the response rate is satisfactory<br>b) The response rate is unsatisfactory, or the comparability between respondents and non-respondents is unsatisfactory.<br>c) No description of the response rate or the characteristics of the responders and the non-responders.	a) independent blind assessment<br>b) record linkage<br>c) self-report *<br>d) no description	a) The statistical test used to analyze the data is clearly described and appropriate, and the measurement of the association is presented, including confidence intervals and the probability level (p value). * b) The statistical test is not appropriate, not described or incomplete.	***
Rajkumari, R;2017	a) truly representative of the average __________(describe) in the community*<br>b) somewhat representative of the average _____ in the community<br>c) selected group of users eg nurses, volunteers<br>d) no description of the derivation of the cohort	a) Justified and satisfactory*<br>b) Not justified.	a) Comparability between respondents and non-respondents characteristics is established, and the response rate is satisfactory<br>b) The response rate is unsatisfactory, or the comparability between respondents and non-respondents is unsatisfactory.<br>c) No description of the response rate or the characteristics of the responders and the non-responders.	a) independent blind assessment<br>b) record linkage<br>c) self-report *<br>d) no description	a) The statistical test used to analyze the data is clearly described and appropriate, and the measurement of the association is presented, including confidence intervals and the probability level (p value). * b) The statistical test is not appropriate, not described or incomplete.	***
Ghaderi, F.; 2017	a) truly representative of the average __________(describe) in the community*<br>b) somewhat representative of the average _____ in the community<br>c) selected group of users eg nurses, volunteers<br>d) no description of the derivation of the cohort	a) Justified and satisfactory*<br>b) Not justified.	a) Comparability between respondents and non-respondents characteristics is established, and the response rate is satisfactory<br>b) The response rate is unsatisfactory, or the comparability between respondents and non-respondents is unsatisfactory.<br>c) No description of the response rate or the characteristics of the responders and the non-responders.	a) independent blind assessment<br>b) record linkage<br>c) self-report *<br>d) no description	a) The statistical test used to analyze the data is clearly described and appropriate, and the measurement of the association is presented, including confidence intervals and the probability level (p value). * b) The statistical test is not appropriate, not described or incomplete.	****
Ayla Acikgoz 2017	a) truly representative of the average __________(describe) in the community<br>b) somewhat representative of the average _____ in the	a) Justified and satisfactory<br>b) Not justified.	a) Comparability between respondents and non-respondents characteristics is established, and the response rate is satisfactory<br>b) The response rate is	a) independent blind assessment<br>b) record linkage	a) The statistical test used to analyze the data is clearly described and appropriate, and the measurement of the association is presented, including confidence intervals and the probability level (p value). * b) The	***

	community*<br>c) selected group of users eg nurses, volunteers<br>d) no description of the derivation of the cohort		unsatisfactory, or the comparability between respondents and non-respondents is unsatisfactory.<br>c) No description of the response rate or the characteristics of the responders and the non-responders.	c) self-report *<br>d) no description	statistical test is not appropriate, not described or incomplete.	
Rumana Akbari M, 2017	a) truly representative of the average __________(describe) in the community<br>b) somewhat representative of the average _____ in the community*<br>c) selected group of users eg nurses, volunteers<br>d) no description of the derivation of the cohort	a) Justified and satisfactory<br>b) Not justified.	a) Comparability between respondents and non-respondents characteristics is established, and the response rate is satisfactory<br>b) The response rate is unsatisfactory, or the comparability between respondents and non-respondents is unsatisfactory.<br>c) No description of the response rate or the characteristics of the responders and the non-responders.	a) independent blind assessment<br>b) record linkage<br>c) self-report *<br>d) no description	a) The statistical test used to analyze the data is clearly described and appropriate, and the measurement of the association is presented, including confidence intervals and the probability level (p value). * b) The statistical test is not appropriate, not described or incomplete.	
Katwal, P.C; 2016	a) truly representative of the average __________(describe) in the community<br>b) somewhat representative of the average _____ in the community*<br>c) selected group of users eg nurses, volunteers<br>d) no description of the derivation of the cohort	a) Justified and satisfactory<br>b) Not justified.	a) Comparability between respondents and non-respondents characteristics is established, and the response rate is satisfactory<br>b) The response rate is unsatisfactory, or the comparability between respondents and non-respondents is unsatisfactory.<br>c) No description of the response rate or the characteristics of the responders and the non-responders.	a) independent blind assessment<br>b) record linkage<br>c) self-report *<br>d) no description	a) The statistical test used to analyze the data is clearly described and appropriate, and the measurement of the association is presented, including confidence intervals and the probability level (p value). * b) The statistical test is not appropriate, not described or incomplete.	***
Aryal, S.; 2016	a) truly representative of the average __________(describe) in the community<br>b) somewhat representative of the average _____ in the community*<br>c) selected group of users eg nurses, volunteers<br>d) no description of the derivation of the cohort	a) Justified and satisfactory<br>b) Not justified.	a) Comparability between respondents and non-respondents characteristics is established, and the response rate is satisfactory<br>b) The response rate is unsatisfactory, or the comparability between respondents and non-respondents is unsatisfactory.<br>c) No description of the response rate or the characteristics of the responders and the non-responders.	a) independent blind assessment<br>b) record linkage<br>c) self-report *<br>d) no description	a) The statistical test used to analyze the data is clearly described and appropriate, and the measurement of the association is presented, including confidence intervals and the probability level (p value). * b) The statistical test is not appropriate, not described or incomplete.	***
Heethal Jaiprakash; 2016	a) truly representative of the average __________(describe) in the community	a) Justified and satisfactory<br>b) Not justified.	a) Comparability between respondents and non-respondents characteristics is established, and	a) independent blind assessment	a) The statistical test used to analyze the data is clearly described and appropriate, and the measurement of the association is presented,	***

	b) somewhat representative of the average _____ in the community*<br>c) selected group of users eg nurses, volunteers<br>d) no description of the derivation of the cohort		the response rate is satisfactory<br>b) The response rate is unsatisfactory, or the comparability between respondents and non-respondents is unsatisfactory.<br>c) No description of the response rate or the characteristics of the responders and the non-responders.	b) record linkage<br>c) self-report *<br>d) no description	including confidence intervals and the probability level (p value). * b) The statistical test is not appropriate, not described or incomplete.	
Reda A. Goweda;2016	a) truly representative of the average __________(describe) in the community*<br>b) somewhat representative of the average _____ in the community<br>c) selected group of users eg nurses, volunteers<br>d) no description of the derivation of the cohort	a) Justified and satisfactory*<br>b) Not justified.	a) Comparability between respondents and non-respondents characteristics is established, and the response rate is satisfactory<br>b) The response rate is unsatisfactory, or the comparability between respondents and non-respondents is unsatisfactory.<br>c) No description of the response rate or the characteristics of the responders and the non-responders.	a) independent blind assessment<br>b) record linkage<br>c) self-report *<br>d) no description	a) The statistical test used to analyze the data is clearly described and appropriate, and the measurement of the association is presented, including confidence intervals and the probability level (p value). * b) The statistical test is not appropriate, not described or incomplete.	****
Chintan Madhusudan Raval; 2016	a) truly representative of the average __________(describe) in the community*<br>b) somewhat representative of the average _____ in the community<br>c) selected group of users eg nurses, volunteers<br>d) no description of the derivation of the cohort	a) Justified and satisfactory*<br>b) Not justified.	a) Comparability between respondents and non-respondents characteristics is established, and the response rate is satisfactory<br>b) The response rate is unsatisfactory, or the comparability between respondents and non-respondents is unsatisfactory.<br>c) No description of the response rate or the characteristics of the responders and the non-responders.	a) independent blind assessment<br>b) record linkage<br>c) self-report *<br>d) no description	a) The statistical test used to analyze the data is clearly described and appropriate, and the measurement of the association is presented, including confidence intervals and the probability level (p value). * b) The statistical test is not appropriate, not described or incomplete.	****
Maryam;2016	a) truly representative of the average __________(describe) in the community<br>b) somewhat representative of the average _____ in the community*<br>c) selected group of users eg nurses, volunteers<br>d) no description of the derivation of the cohort	a) Justified and satisfactory<br>b) Not justified.	a) Comparability between respondents and non-respondents characteristics is established, and the response rate is satisfactory<br>b) The response rate is unsatisfactory, or the comparability between respondents and non-respondents is unsatisfactory.<br>c) No description of the response rate or the characteristics of the responders and the non-responders.	a) independent blind assessment<br>b) record linkage<br>c) self-report *<br>d) no description	a) The statistical test used to analyze the data is clearly described and appropriate, and the measurement of the association is presented, including confidence intervals and the probability level (p value). * b) The statistical test is not appropriate, not described or incomplete.	***

Supplementary Table 2- Lifestyle factors associated with menstrual disturbances amongst female medical students

Life-style factors	Author, Year
Smoking	Alkhamis, F. 2021; Shah, RS. 2020; Minichil, W. 2020; Ozder, A. 2020; Acikgoz, A. 2017; Katwal, P.C. 2016; Goweda A. R. 2016 (n=7)
Tea	Kanti, V. 2020; Ozder, A. 2020 (n=2)
Coffee intake	Alkhamis, F. 2021; Hashim, R.T. 2021; Kanti, V. 2020; Ozder, A. 2020; Sharma, P. 2018; Maryam, M. 2016 (n=6)
Lack of exercise	Alkhamis, F. 2021; Kushwaha, R.P. 2021; Kanti, V. 2020; Bilir, E. 2020; Verma I. 2020; Nama S. 2020; Ghaderi, F. 2017; Katwal, P.C. 2016; Raval, C.M. 2016; Sharma, P. 2018 (n=10)
Underweight/Obesity/ high BMI	Al-Shahrani, A. 2021; Kanti, V. 2020; Nama S. 2020; Rajkumari, R. 2017; Rumana Akbari, M. 2017; Katwal, P.C. 2016; Jaiprakash, H. 2016; Raval, C.M. 2016 (n=8)
Unhealthy/Junk/High-salt/ food or diet and/or sweets	Alkhamis, F. 2021; Al-Shahrani, A. 2021; Kanti, V. 2020; Acikgoz, A. 2017; Rumana Akbari, M. 2017 (n=5)
Family history	Katwal, P.C. 2016; Jaiprakash, H. 2016; Raval, C.M. 2016; Maryam, M. 2016 (n=4)
Psychologic or other diseases/ stress	Minichil, W. 2020; Ozder, A. 2020; Verma I. 2020; Majeed-Saidan, MMA. 2020; Nama S. 2020; Rajkumari, R. 2017; Acikgoz, A. 2017; Katwal, P.C. 2016; Goweda A. R. 2016; Aryal, S. 2016; Maryam, M. 2016 (n=11)
Miscellaneous/Non-specific	Zalat, MM. 2019; Yadav, B. 2019; Shamnani, G. 2018 (n=3)
